# Estrogen-induced circRNA, circPGR, functions as a ceRNA to promote estrogen receptor-positive breast cancer cell growth by regulating cell cycle-related genes

**DOI:** 10.7150/thno.45302

**Published:** 2021-01-01

**Authors:** Lei Wang, Jia Yi, Ling-yun Lu, Yue-ying Zhang, Lan Wang, Guo-sheng Hu, Yi-chen Liu, Jian-cheng Ding, Hai-feng Shen, Fang-qing Zhao, Hai-hua Huang, Wen Liu

**Affiliations:** 1 Fujian Provincial Key Laboratory of Innovative Drug Target Research, School of Pharmaceutical Sciences, Xiamen University, Xiang'an South Road, Xiamen, Fujian 361102, China.; 2 State Key Laboratory of Cellular Stress Biology, School of Pharmaceutical Sciences, Xiamen University, Xiang'an South Road, Xiamen, Fujian 361102, China.; 3 Department of Orthopedics, The Fifth Hospital of Xiamen, Xiamen, Fujian 361101, China.; 4 Department of Pathology, The Second Affiliated Hospital, Shantou University Medical College, Dongxia North Road, Shantou, Guangdong 515041, China.; 5 Computational Genomics Lab, Beijing Institutes of Life Science, Chinese Academy of Sciences, Beijing 100101, China.

**Keywords:** estrogen and estrogen receptor, circRNA, miRNA, cell cycle, ER-positive breast cancer

## Abstract

Estrogen and estrogen receptor (ER)-regulated gene transcriptional events have been well known to be involved in ER-positive breast carcinogenesis. Meanwhile, circular RNAs (circRNAs) are emerging as a new family of functional non-coding RNAs (ncRNAs) with implications in a variety of pathological processes, such as cancer. However, the estrogen-regulated circRNA program and the function of such program remain uncharacterized.

**Methods:** CircRNA sequencing (circRNA-seq) was performed to identify circRNAs induced by estrogen, and cell proliferation, colony formation, wound healing, transwell and tumor xenograft experiments were applied to examine the function of estrogen-induced circRNA, circPGR. RNA sequencing (RNA-seq) and ceRNA network analysis wereperformed to identify circPGR's target genes and the microRNA (miRNA) bound to circPGR. Anti-sense oligonucleotide (ASO) was used to assess circPGR's effects on ER-positive breast cancer cell growth.

**Results:** Genome-wide circRNA profiling by circRNA sequencing (circRNA-seq) revealed that a large number of circRNAs were induced by estrogen, and further functional screening for the several circRNAs originated from PGR revealed that one of them, which we named as circPGR, was required for ER-positive breast cancer cell growth and tumorigenesis. CircPGR was found to be localized in the cytosol of cells and functioned as a competing endogenous RNA (ceRNA) to sponge miR-301a-5p to regulate the expression of multiple cell cycle genes. The clinical relevance of circPGR was underscored by its high and specific expression in ER-positive breast cancer cell lines and clinical breast cancer tissue samples. Accordingly, anti-sense oligonucleotide (ASO) targeting circPGR was proven to be effective in suppressing ER-positive breast cancer cell growth.

**Conclusions:** These findings reveled that, besides the well-known messenger RNA (mRNA), microRNA (miRNA), long non-coding RNA (lncRNA) and enhancer RNA (eRNA) programs, estrogen also induced a circRNA program, and exemplified by circPGR, these estrogen-induced circRNAs were required for ER-positive breast cancer cell growth, providing a new class of therapeutic targets for ER-positive breast cancer.

## Introduction

The first observation suggesting that eukaryotic RNAs can exist in circular form was made more than 40 years ago by transmission electron microscopy 1. Ten years later, circular RNAs (circRNAs) were identified on a few genes and thought to be by-products of mis-splicing or splicing errors 2-5. It was not until recently that, with the advances in high-throughput sequencing techniques and computational approaches, circRNAs are emerging as a new family of functional non-coding RNAs (ncRNAs) that are highly present and conserved, and often exhibit tissue- and developmental stage-specific expression in the eukaryotic transcriptome 6-11.

CircRNAs are distinct from other RNA species in that the otherwise free 5' and 3' ends are covalently linked, resulting in a closed loop structure without a 5' cap structure or 3' polyadenylated tail 12. The closed loop structure exempts circRNAs from endonuclease degradation, and therefore they are highly stable *in vivo* compared with their linear counterparts. Although the majority of circRNAs are derived from coding exons (CDS), which may consist of a single or multiple exons, circRNAs can also arise from introns, intergenic regions, 5' and 3' untranslated regions (UTRs) and from locations antisense to known transcripts 13-20. The sites at which the circRNA ends are joined are often flanked by canonical splice signals, suggesting that the spliceosome is involved in the production of circRNAs 21. Two mechanisms, exon skipping and back-splicing, are proposed to be involved in the formation of exonic circRNAs 10. The lariat structure formed by exon skipping enables circularization, whereas back-splicing involves an upstream 3' splice site (donor) joining to a downstream 5' splice site (acceptor), which is in opposite to linear splicing where a downstream 3' splice site is joined to an upstream 5' splice site. The formation of circRNAs is regulated by both cis-elements and trans-factors 7. Recent studies have shown that exon circularization is facilitated by surrounding complementary sequences 22-24, such as inverted repeated Alu pairs, and specific protein factors, such as RNA editing enzyme ADAR1 24, the alternative splicing factor Quaking 25 and RBM20 26.

Once produced, exon-intron circRNAs (circRNAs with retained introns) may reside in the nucleus, whereas majority of the exonic circRNAs are found to localize in the cytoplasm 19, 20, 27. It is known that circRNAs play important roles in both transcriptional and post-transcriptional regulation, and multiple mechanisms through which circRNAs exert their functions are existed. Cytoplasmic exonic circRNA can act as microRNA (miRNA) sponge to inhibit the functions of miRNAs it binds to, and thus regulating gene expression, which was exemplified by circRNA ciRS-7 (also called CDR1as), a circRNA harboring more than 70 conventional miR-7-binding sites 15, 28. It was shown that nuclear-localized exon-intron circRNAs with retained introns promoted transcription of their parental gene through interactions with RNA polymerase II machinery 19, 20. CircRNAs can also function in gene regulation by competing with linear splicing. A circRNA produced from muscleblind (mbl) was shown to be involved in the auto-regulation of this RNA-binding protein 29. Additionally, circRNAs might function as scaffold for protein-protein interactions to occur, modulating protein functions. For instance, Foxo3 circRNA inhibits cell cycle progression via forming ternary complexes with p21 and CDK2 30, ANRIL circRNA modulates ribosomal RNA (rRNA) maturation and atherosclerosis through its binding with pescadillo homologue 1 (PES1), an essential 60S-preribosomal assembly factor 31 and circACC1 has been shown that it can directly binds to both AMPKβ and γ Subunits, facilitating AMPK holoenzyme assembly, stability and activity 32.

Many circRNAs have been found to be dynamically regulated and functional in physiological process of aging 33, 34 and development 16, 35-37. In addition, a growing number of studies have demonstrated that circRNAs are tightly associated with pathological processes, such as cancer, heart diseases, neurological disorders, diabetes and atherosclerosis, with implications in diagnosis and treatment of diseases 31, 38-41. A novel fusion circRNA (F-circM9) was found to be only present in leukemia cells where MLL/AF9 gene translocation occurs, and expression of F-circM9 promotes leukemia cell proliferation 42; circPTCH1 promoted renal cell carcinoma (RCC) metastasis via the miR-485-5p/MMP14 axis and activation of the EMT process and targeting circPTCH1 may represent a promising therapeutic strategy for metastatic RCC 43. CircRNA cSMARCA5 was found to be down-regulated in HCC and associated with poor prognosis 44; CircPVT1 was reported to be highly expressed in head and neck squamous cell carcinoma, which affected the development of head and neck squamous cell carcinoma 45; Circ-Ccnb1 producing from CCNB1 was specifically down-regulated in breast cancer and functioned as a tumor suppressor, with that stable over-expression or direct injection of circ-Ccnb1 overexpression plasmid could significantly inhibit tumor growth 46; CircEPSTI1 acted as a miRNA sponge to sponge miR-4753 and miR-6809, up-regulating BCL11A expression and promoting triple negative breast cancer (TNBC) cell proliferation 47; Hypoxia induced exosomal circRNA, circ-133 was found to promote metastasis of colorectal cancer 48. CircRNAs might also serve as diagnosis biomarkers. For instance, circRNAs were found to be widely distributed in hepatocarcinoma (HCC) exosomes. Due to the covalent closed-loop structure of circRNA, these exosomal circRNAs may become biomarkers for cancer “liquid biopsy” 49.

Antisense oligonucleotides (ASOs) are a class of small-sized, single-stranded deoxyribonucleotide analogue that are designed to bind to the complementary RNA in both nucleus and cytosol of a cell 50. ASOs have been not only broadly used in protein or RNA biology 51, but also served as a highly promising therapeutic strategy for many diseases 52-54. Recent study has shown that ASOs can be used to investigate a circular but not linear RNA-mediated function for its encoded gene locus 55, which makes back-splicing-junction (BSJ) targeting-ASOs a highly promising and specific strategy for circRNA targeting.

Estrogen (17β-estradiol, E_2_) plays a critical role in a variety of biological processes, such as the reproductive system, the central nervous system, the skeleton, the circulatory system and the immune system 56-58. The biological actions of estrogen are mediated through estrogen receptors (ERα and ERβ), which mainly function in the nucleus as estrogen-dependent transcription factors 58. In conjunction with other coactivator protein complexes, estrogen-bound estrogen receptor binds to estrogen response elements (EREs) and activates transcription of genes with implications in proliferation and differentiation in various tissues, including breast epithelial cells 59. In addition to activate coding gene program, estrogen/ER has also been reported to activate the transcription of non-coding RNAs, including microRNAs (miRNAs) 60, long non-coding RNAs (lncRNAs) 61 and enhancer RNAs (eRNAs) 62. Accordingly, aberrant estrogen responses are associated with a variety of hormone-dependent diseases, particularly breast cancer. 70% of breast carcinomas are ER positive and estrogen dependent. Estrogen antagonists can block mitogenic signaling induced by estrogen 63. In fact, selective estrogen receptor modulators (SERMs) represent the frontline interventions for the treatment of breast cancers, although in some cases, patients develop SERM resistance 64.

Despite the prevalent role of both estrogen and circRNA in breast cancer development, whether an estrogen-induced circRNA program exists and the function of such program remain uncharacterized. In this study, we generated ribo-minus and RNase R-treated RNA sequencing data from estrogen-stimulated MCF7 breast cancer cells, and identified a large number of estrogen-induced circRNAs, among which circPGR was found to promote ER-positive breast cancer cell growth and tumorigenesis by sponging miR-301a-5p to regulate the expression of multiple cell cycle genes. CircPGR was highly and specifically expressed in ER-positive breast cancer cell lines and clinical breast cancer tissue samples. Targeting circPGR by ASO was effective in suppressing ER-positive breast cancer cell growth.

## Results

### CircRNAs are prevalent in MCF7 ER-positive breast cancer cells

**Figure 1:**
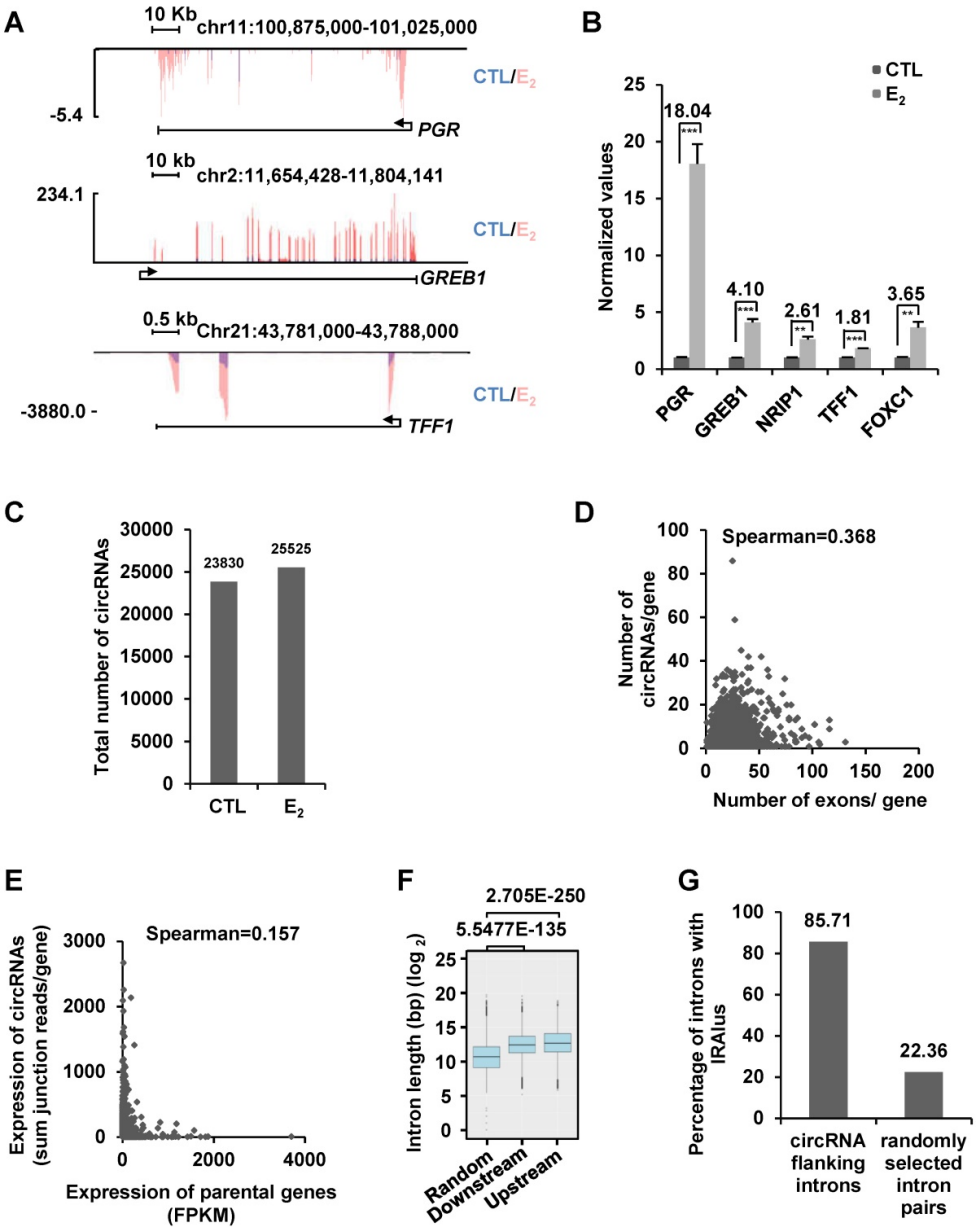
** A large number of circRNAs are detected by circRNA-seq in MCF7 cells.** (**A**) MCF7 cells were cultured in stripping medium for three days and treated with or without estrogen (E_2_, 10^-7^ M, 6 h) followed by RNA-seq analysis, and genome browser views of selected genes as indicated were shown. Blue: control (CTL); Red: estrogen (E_2_). (**B**) RNA samples as described in (A) were subjected to RT-qPCR analysis to examine the expression of *PGR*, *GREB1*, *NRIP1*, *TFF1* and *FOXC1*. Fold change by estrogen treatment was shown as indicated. Data shown was the relative fold change compared to control samples (CTL) after normalization to actin (± s.e.m, **P < 0.01, ***P < 0.001). (**C**) RNA samples as described in (A) were subjected to circRNA-seq. Total number of circRNAs predicted from circRNA-seq were shown (unique junction reads ≥ 2). (**D**) Correlation between the number of exons a gene has and circRNAs originated from that gene was shown. (**E**) Correlation between the expression of parental gene (FPKM) and circRNA originated from the same gene region (average junction reads/gene) was shown. (**F**) Box plot showing the length of randomly selected introns or introns flanking, upstream and downstream, circRNA-producing regions. Median: 1,639 bp (random); 5,526 bp (downstream); 6,578 bp (upstream). bp: base pair. (**G**) Percentage of randomly selected introns or introns flanking circRNA-producing regions containing inverted repeated Alu pairs was shown.

To examine whether estrogen can induce the expression of circRNAs, we performed ribo-minus and RNase R-treated RNA sequencing (circRNA-seq) in MCF7 cells cultured in stripping medium and then treated with or without estrogen. To assess the estrogen effects, we also performed regular RNA sequencing (without RNase R treatment) in parallel by using the same batch of samples. As expected, estrogen induced the expression of a substantial list of coding genes as reported previously, such as *PGR*, *GREB1*, *TFF1*, *NRIP1*, and *FOXC1*, which was further validated by RT-qPCR analysis, suggesting that the estrogen treatment was effective (Figure 1A-B and Figure S1A). We then analyzed our circRNA-seq by CIRI2 65 and found that 23,830 and 25,525 circRNAs were present in control and estrogen-treated cells, respectively (unique junction reads ≥ 2) (Figure 1C and Figure S1B). The junction reads for circRNAs identified in two biological repeats were highly correlated, supporting the reproducibility of our circRNA-seq (Figure S1C).

We first focused on analyzing those 23,830 circRNAs identified in control cells without estrogen treatment. A large set of circRNAs were found to be highly expressed in MCF7 cells, with nearly 900 circRNAs had more than 50 back splicing junction (BSJ) reads detected under current sequencing depth (~28.5 million reads per sample) (Figure S1D). Genomic distribution analysis of these 23,830 circRNAs revealed that they were originated from 5,933 gene regions (5' UTR, exons, introns and 3' UTR) and 1,330 intergenic regions. There were often multiple circRNAs producing from the same gene region, exemplified by TRIM37 gene region, in which 86 circRNAs in total were found (Table S1). The number of circRNAs produced from a gene appeared to be poorly correlated with the number of exons that gene has (Figure 1D). For instance, only one circRNA was found to be originated from TTN, which itself has 363 exons (Figure S1E). In contrast, NEAT1, a single exon-containing gene, was found to have 17 circRNAs produced from this gene region (Figure S1F). The correlation between the expression of circRNAs and parental genes was found to be extremely poor (Figure 1E). For instance, KRT8 was the second highest expressed gene (FPKM 3,391.67) among genes with at least one circRNA, whereas both of the two circRNAs originated from this gene were found to have only 2 junction reads. In contrast, ATXN7, a gene expressed at relatively low levels (FPKM 4.28), was found to have the most highly expressed circRNA with 2,575 junction reads (Table S1). There were also a number of genes not expressing (n=30), at least at current sequencing depth, in MCF7 cells but with detectable circRNAs. For instance, MIA2, a gene not expressing in MCF7 cells, was found to have 5 circRNAs produced from the same gene region with junction reads ranging from 2 to 99 (Table S1). It has been reported that the length of introns flanking genomic regions that produce circRNAs is in general longer than those do not. Similarly, the length of introns flanking the circRNAs identified in our circRNA-seq analysis was significantly longer than that of randomly selected introns (Figure 1F). The circularization of circRNAs is often mediated through complementary sequences embedded in flanking introns, such as inverted repeated Alu pairs (IRAlu) 13, 21. Searching for Alu repeats in introns flanking circRNAs we identified revealed that nearly 86% of flanking introns had inverted repeated Alu pairs, which was only 22% for randomly selected introns (Figure 1G). Finally, the identity of randomly selected circRNAs was confirmed by Sanger sequencing (Figure S1G-J). The analysis described above was also performed for those 25,524 circRNAs identified in estrogen-treated cells, and similar conclusions were reached. Specifically, the number of circRNAs produced from a gene was poorly correlated with the number of exons that gene has, the expression level of circRNAs were also poorly correlated with that of their linear counterparts, and the length of introns and the percentage of introns flanking circRNAs bearing inverted repeated Alu repeats were also significantly different from that of randomly selected introns (data not shown).

We also analyzed our circRNA-seq data by using two other pipelines, find_circ 66 and CIRCexplorer2 67, and compared the circRNAs identified to those of CIRI2. CIRI2 identified the most, whereas CIRCexplorer2 identified the least number of circRNAs, and around 59% circRNAs identified by CIRCexplorer2 were also predicted by the other two pipelines (junction reads ≥ 2), which was consistent with previous reports comparing the reproducibility of different pipelines in identifying circRNAs (Figure S2A) 9, 68, 69. Interestingly, when we increased the cutoff of junction reads, the percentage of circRNAs commonly predicted by the three pipelines also increased, suggesting that highly expressed circRNAs could be easily identified by all three pipelines (Figure S2A-C). Similar conclusions could be drawn when we repeated the analysis shown in Figure 1D-G based on the circRNAs predicted by find_circ or CIRCexplorer2 (Figure S2D-K). Based on our extensive validation of circRNAs predicted by the three pipelines, we found that the false positive rate for all of them was pretty low (i.e, as long as one circRNA was identified by one pipeline, this circRNA could often be validated). We therefore chose to focus on the results from CIRI2, which predicted the largest number of circRNAs.

### A large number of circRNAs are induced by estrogen

**Figure 2:**
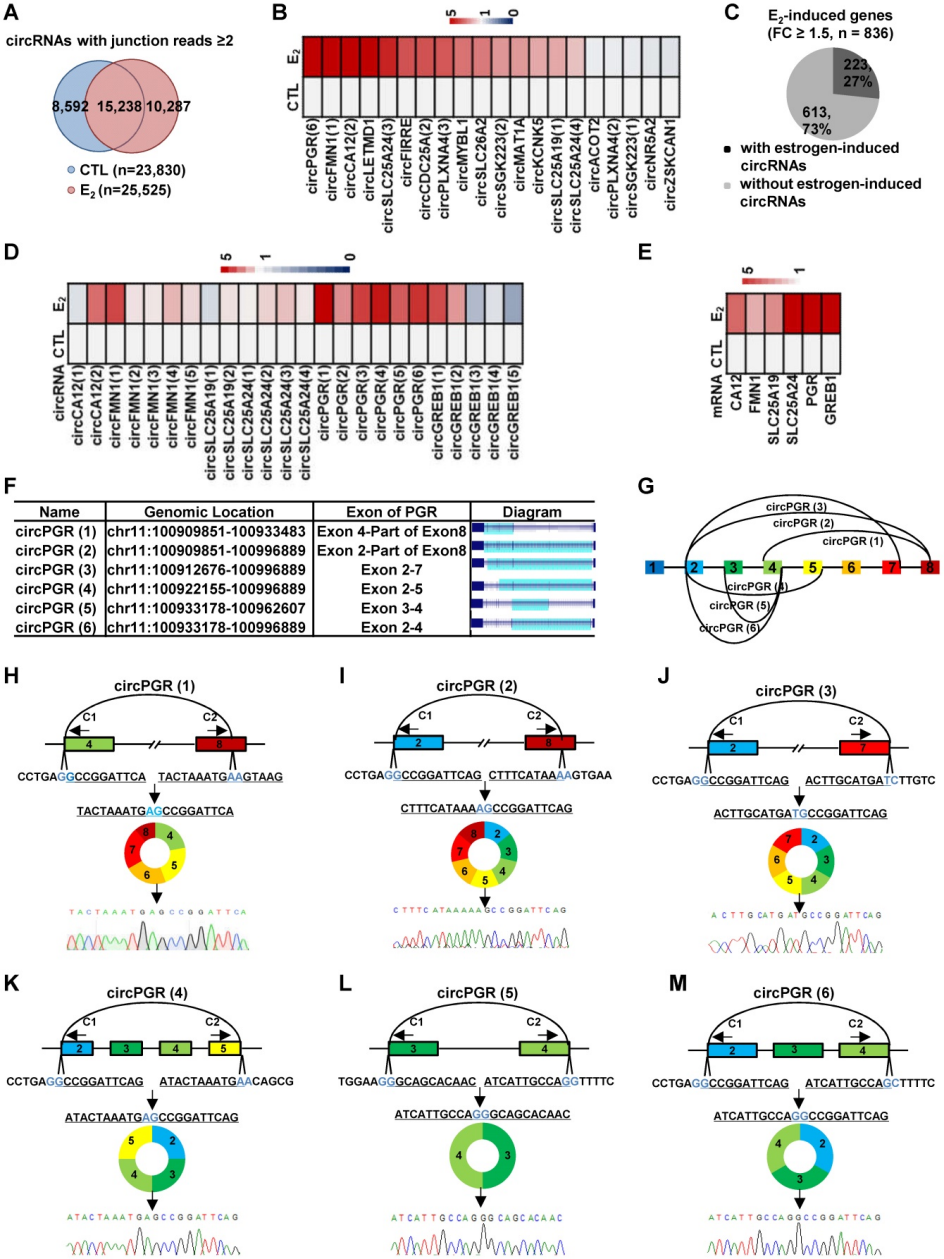
** A large number of circRNAs are induced by estrogen.** (**A**) Venn diagram showing overlapping between circRNAs predicted in both control (CTL) and estrogen (E_2_)-treated conditions (junction reads ≥ 2, FC ≥ 2). (**B**) RNA samples as described in Figure 1A were subjected to RT-qPCR analysis using divergent primer sets flanking circRNA junction regions to examine the expression of estrogen-induced circRNAs. Genomic location information on all circRNAs shown could be found in Supplementary Table 1. (**C**) Estrogen-induced genes (FC ≥ 1.5) were determined based on regular RNA-seq performed in parallel, and estrogen-induced gene regions with or without detectable estrogen-induced circRNA were shown by pie chart. (**D**) RNA samples as described in Figure 1A were subjected to RT-qPCR analysis using divergent primer sets flanking circRNA junction regions to examine the expression of circRNAs generated from estrogen-induced genes as indicated. Genomic location information on all circRNAs shown could be found in Supplementary Table 1. (**E**) RNA samples as described in Figure 1A were subjected to RT-qPCR analysis to examine the expression of estrogen-induced parental genes corresponding to those circRNAs shown in (D). (**F**) Genomic location and schematic representation of the six circRNAs, circPGR (1) to circPGR (6), originated from *PGR* gene. CircRNA was highlighted in light blue. (**G**) Diagram showing the six circRNAs originated from *PGR* gene. (**H-M**) RNA samples from estrogen-treated MCF7 cells as described in Figure 1A was subjected to reverse transcription, and standard PCR was performed by using divergent primer sets flanking the junction regions of circPGRs, followed by Sanger sequencing. Sequence flanking junction regions was shown. Junction sites were highlighted in light blue. Sanger sequencing histogram was shown at the bottom. C1: convergent primer 1; C2: convergent primer 2.

We next asked whether estrogen could alter the circRNA program in MCF7 cells by comparing circRNAs identified in estrogen-treated cells to that of control cells. There were 10,287 and 8,592 circRNAs found to be induced or repressed by estrogen treatment, respectively (junction reads ≥ 2, fold change ≥ 2) (Figure 2A)**.** When we set the cutoff of junction reads at 5 or 10, the percentage of circRNAs that were specifically presented in control or estrogen-treated conditions decreased (Figure S3A-B). In the current study, we focused on studying the function and molecular mechanisms of estrogen-induced circRNAs in ER-positive breast cancer. We sought to validate estrogen-induced circRNAs by RT-qPCR analysis. Out of the 25 estrogen-induced circRNAs randomly chosen for validation (FC ≥ 2), the primers designed for 5 of them were not suitable for RT-qPCR analysis due to multiple PCR products could be observed, which most likely were generated from alternative circularization for the targeted regions. For the rest of 20 circRNAs examined, 15 were successfully validated to be induced by estrogen (Figure 2B and Figure S3C). We therefore estimated that 75% of estrogen-induced circRNAs identified by our circRNA-seq could be validated. During the course of validation, we noticed that the parental genes (linear counterparts) of many of the estrogen-induced circRNAs were also induced by estrogen. Specifically, 22% of estrogen-induced genes were found to be associated with estrogen-inducible circRNAs (Figure 2C). Furthermore, multiple estrogen-induced circRNAs were often observed to be produced from the same estrogen-induced gene. We therefore asked whether these circRNAs produced from the same gene were similarly induced by estrogen. To this end, circRNAs produced from estrogen-induced genes, such as those from* CA12*, *FMN1*, *SLC25A19*, *SLC25A24*, *PGR* and *GREB1*, were subjected to validation. It was found that these circRNAs were not uniformly induced by estrogen treatment, and some of them were even not inducible (Figure 2D). As expected, the parental genes were validated to be induced by estrogen (Figure 2E). The 6 circRNAs, which we named as circPGR (1) to circPGR (6), produced from *PGR* gene caught our attention due to the fact that they were all inducible by estrogen and among the top most inducible circRNAs (Figure 2D, 2F-G), which were further validated by Sanger sequencing (Figure 2H-M).

### Estrogen-induced circRNA, circPGR, promotes ER-positive breast cancer cell growth and tumorigenesis

**Figure 3:**
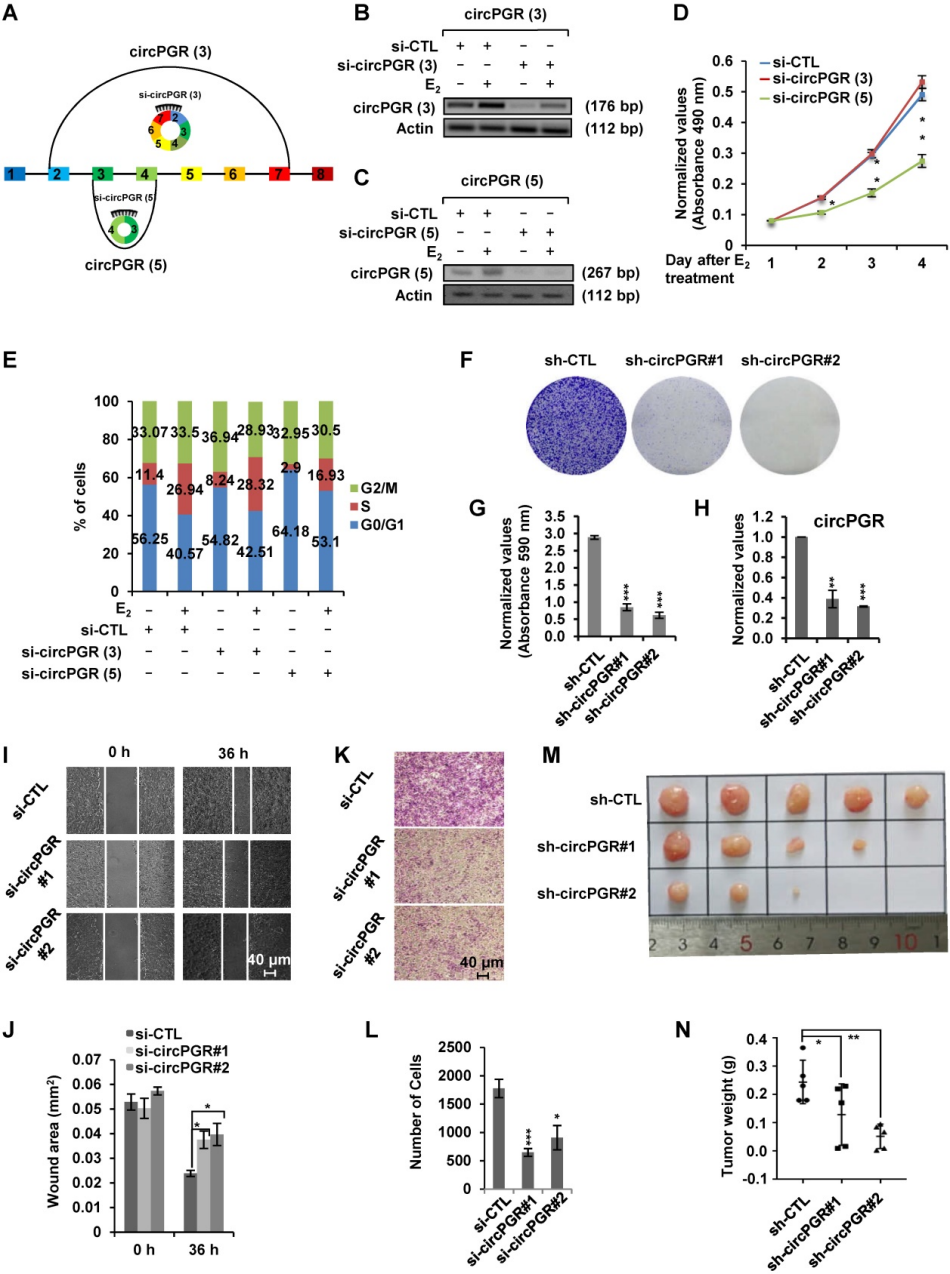
** Knockdown of Estrogen-induced circPGR inhibits ER-positive breast cancer cell growth and tumorigenesis.** (**A**) Diagram showing the two circPGRs, circPGR (3) and circPGR (5), with designable siRNAs targeting junction regions. (**B, C**) MCF7 cells cultured in stripping medium were transfected with control siRNA (si-CTL) or siRNA specifically targeting circPGR (3) (si-circPGR (3)) (B) or circPGR (5) (si-circPGR (5)) (C) and treated with or without estrogen (E_2_, 10^-7^ M, 6 h) followed by standard PCR and DNA electrophoresis to visualize the knockdown efficiency of si-circPGR (3) and si-circPGR (5). Actin was served as a loading control. The size of PCR products was shown as indicated. bp: base pair. (**D, E**) MCF7 cells cultured in stripping medium were transfected with control siRNA (si-CTL) or siRNA specifically targeting circPGR (3) (si-circPGR (3)) or circPGR (5) (si-circPGR (5)) and treated with or without estrogen (E_2_, 10^-7^ M, 6 h) for duration as indicated, followed by cell proliferation assay (D) and FACS analysis (E) (± s.e.m., *P < 0.05, **P < 0.01). (**F**) MCF7 cells were infected with lenti-viral control shRNA (sh-CTL) or two independent shRNAs specifically targeting circPGR (sh-circPGR#1 and sh-circPGR#2) followed by colony formation assay. (**G**) Quantification of the crystal violet dye as shown in (F) (± s.e.m., ***P < 0.001). (**H**) MCF7 cells as described in (F) was subjected to RNA extraction and RT-qPCR analysis to examine the expression of circPGR. Data shown was the relative fold change compared to control samples (sh-CTL) after normalization to actin (± s.e.m., **P < 0.01, ***P < 0.001). (**I, K**) MCF7 cells transfected with control siRNA (si-CTL) or two independent siRNAs specifically targeting circPGR (si-circPGR#1 and si-circPGR#2) for 48 h were both re-seeded at full confluence and then subjected to wound healing (I) or trans-well (K) assay. (**J, L**) Quantification of wound closure (J) and number of colonies (L) as shown in (I) and (K), respectively (± s.e.m., *P < 0.05, ***P < 0.001). (**M**) MCF7 cells infected with lenti-viral control shRNA (sh-CTL) or two independent shRNAs specifically targeting circPGR (sh-circPGR#1 and sh-circPGR#2) were injected subcutaneously into female BALB/C nude mice for xenograft experiments. (**N**) Tumor weight as shown in (M) (± s.e.m., *P < 0.05, **P < 0.01).

As many of the estrogen-induced genes are functional in promoting ER-positive breast cancer cell growth, we next sought to examine whether circPGRs are also able to do so. To this end, we attempted to design siRNA specifically targeting the back splicing junction region of each individual circPGR. Out of the 6 circRNAs, only the junction regions of 2 of them, circPGR (3) and circPGR (5), were suitable for siRNA design. Indeed, only siRNAs targeting circPGR (3) and circPGR (5) were found to be effective in knocking down their targeted circPGRs (Figure 3A-C). We then tested the effects of circPGR (3) and circPGR (5) on MCF7 cell growth by transfecting MCF7 cells cultured in stripping medium with control siRNA or siRNA specifically targeting circPGR (3) or circPGR (5), and then treated with or without estrogen followed by cell proliferation assay, finding that knockdown of circPGR (5) significantly attenuated estrogen-induced cell proliferation, whereas knockdown of circPGR (3) had no significant impact (Figure 3D). We noticed that knockdown of circPGR also led to downregulation of its parental gene *PGR* (Figure S4A). To exclude the possibility that the effects of circPGR (5) on cell proliferation was due to downregulation of *PGR*, we knocked down PGR in MCF7 cells and found that PGR had no significant effects on cell proliferation (Figure S4B-C). FACS analysis revealed that cells were arrested in G_1_ phase when circPGR (5) was knocked down (Figure 3E). For simplicity, we thus named circPGR (5) as circPGR. The effects of circPGR on cell growth were further demonstrated by colony formation assay using two independent shRNAs targeting circPGR (Figure 3F-H). Knockdown of circPGR was also found to affect cell migration and invasion as shown by wound healing assay (Figure 3I-J) and trans-well assay (Figure 3K-L). To confirm circPGR's effects, overexpression of circPGR was found to promote MCF7 cell growth, cell cycle progression, colony formation, cell migration, and cell invasion (Figure S5A-I). Furthermore, knockdown of circPGR was found to inhibit (Figure S6A-H), whereas overexpression of circPGR promote (Figure S7A-H) cell cycle progression, colony formation, cell migration, and cell invasion in another ER-positive cell line, T47D. To test circPGR's effects on tumor growth *in vivo*, we injected nude mice subcutaneously with MCF7 cells infected with lentivirus expressing control shRNA or shRNA targeting circPGR, and then exogenous administration of estrogen to sustain the growth of tumor in mice. CircPGR knockdown significantly attenuated estrogen-induced tumorigenesis (Figure 3M-N). Taken together, our data suggested that circPGR is required for ER-positive breast cancer cell growth and tumorigenesis.

### CircPGR is stably localized in the cytosol of cells

**Figure 4:**
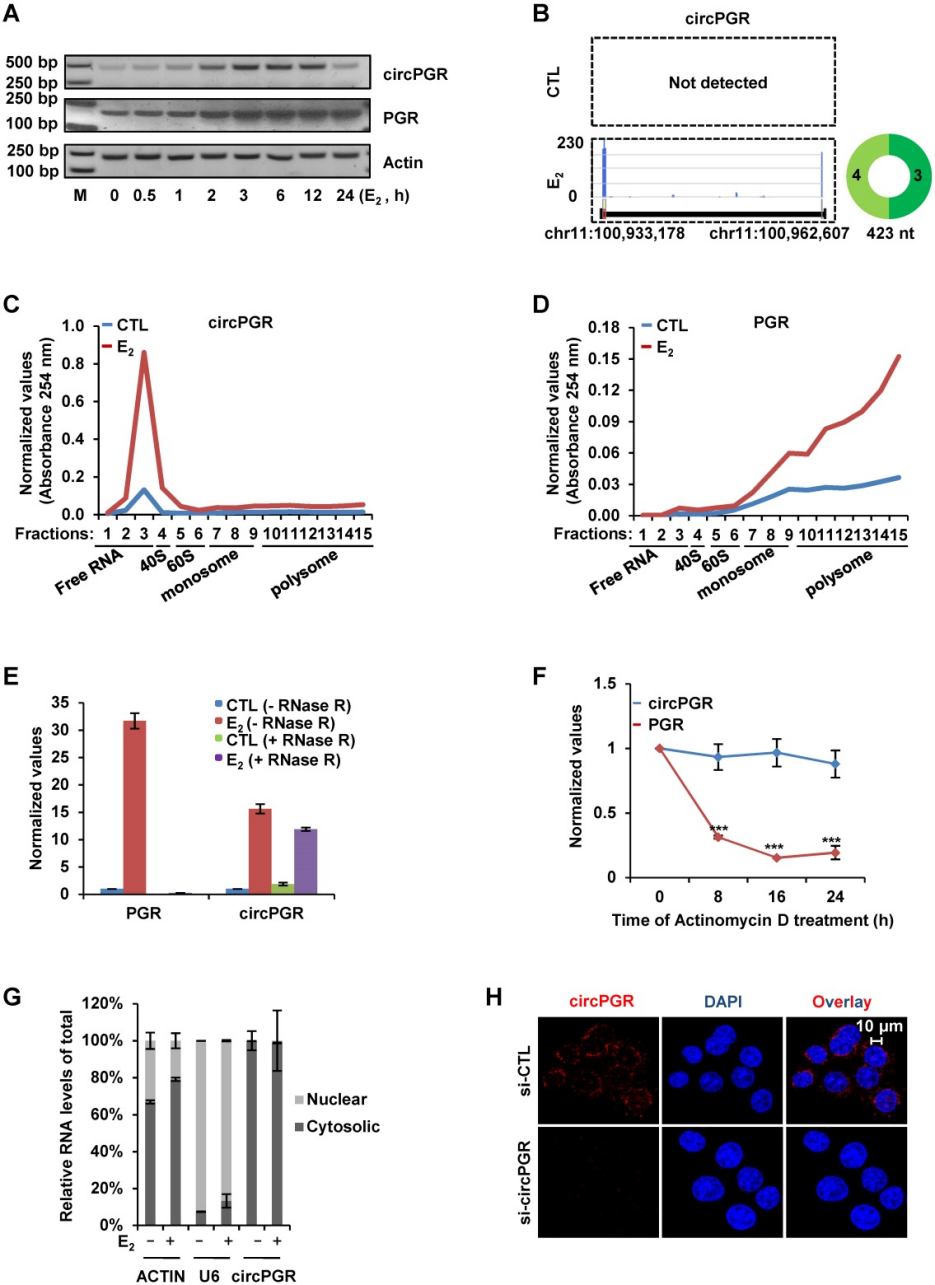
** CircPGR is stably localized in the cytosol of cells.** (**A**) MCF7 cells cultured in stripping medium and treated with or without estrogen (E_2_, 10^-7^ M) for duration as indicated were subjected to RNA extraction, reverse transcription and standard PCR by using primer sets specifically targeting circPGRs or PGR. The resultant PCR products were separated by DNA electrophoresis. Actin was served as a loading control. DNA marker (M) was shown as indicated. bp: base pair. (**B**) Prediction and visualization of full length circPGR expression in both control (CTL) and estrogen (E_2_)-treated conditions by CIRI-full and CIRI-vis, respectively, based on circRNA-seq as described in Figure 1C. (**C, D**) MCF7 cells cultured in stripping medium and treated with or without estrogen (E_2_, 10^-7^ M, 24 h) were subjected to polysome profiling and the resultant fractions were subjected to RNA exaction and RT-qPCR analysis to examine the expression of circPGR (C) and PGR (D). Fractions 1 to 3: free RNA (unbound with ribosome); Fraction 4: 40S (40S ribosomal subunit); Fractions 5 and 6: 60S (60S ribosomal subunit); Fractions 7 to 9: monosome; Fractions 10 to 15: polysome. (**E**) Total RNAs extracted from MCF7 cells cultured in stripping medium and treated with or without (E_2_, 10^-7^ M, 6 h) were incubated with or without RNase R (10 units/μg RNA) at 37 °C for 1 h, followed by RT-qPCR analysis to examine the expression of PGR or circPGR. (**F**) Estrogen-treated MCF7 cells were added with Actinomycin D (10 μg/ml) for duration as indicated, followed by RT-qPCR analysis to examine the expression of PGR or circPGR (± s.e.m., ***P < 0.001). (**G**) MCF7 cells cultured in stripping medium and treated with or without estrogen (E_2_, 10^-7^ M, 6 h) were subjected to cellular fractionation followed by RNA extraction and RT-qPCR analysis to quantify the amount of circRNA in both nucleus and cytosol of the cells. ACTIN and U6 snoRNA were served as purity control for cytosolic and nuclear fractions, respectively. (**H**) RNA-FISH analysis was performed in MCF7 cells transfected with si-CTL or si-circPGR and treated with estrogen (E_2_, 10^-7^ M, 6 h). Red: circPGR; Blue: DAPI.

We next sought to characterize circPGR to see its induction kinetics by estrogen, length, coding potential, cellular localization and stability. Firstly, the kinetics of circPGR's induction by estrogen was found to be similar as its linear counterpart *PGR* in both MCF7 and T47D cells (Figure 4A and Figure S8A). Our previous study indicated that ERα was recruited to the distal enhancer of PGR in the presence of estrogen to regulate *PGR* expression in MCF7 cells (Figure S8B) 70. The expression of circPGR was shown to be dependent on ERα as ICI 182,780 (Fulvestrant), an ERα degrader, significantly attenuated the induction of circPGR (Figure S8C-D). Analyzing circRNA-seq by CIRI-full 65 revealed that circPGR was the full length of exon 3 and 4 from *PGR* gene without any retained intron sequence (Figure 4B). As more and more circRNAs were shown to actually encode microproteins, we also examined whether circPGR has coding potential by performing polysome profiling in control and estrogen-treated MCF7 cells. It was found that circPGR was largely associated with ribosome-free fractions, indicating that it was truly a non-coding RNA (Figure 4C). As expected, its linear counterpart *PGR* was found to be associated with polysomes (Figure 4D). The 5' and 3' ends of circRNAs are covalently linked, resulting in a closed loop structure, which was believed to resist to RNase R digestion and therefore makes circRNAs more stable than their linear counterparts. Indeed, when we digested total RNA extracted from MCF7 cells treated with or without estrogen with RNase R, *PGR* was nearly undetectable while circPGR exhibited resistance to RNase R digestion (Figure 4E). We also compared the stability of circPGR to that of *PGR* by treating MCF7 cells with Actinomycin D, an inhibitor of transcription, and found that circPGR was much more stable than *PGR* (Figure 4F). Next, we examined the cellular localization of circPGR by performing cellular fractionation followed by RNA extraction and RT-qPCR analysis. It was found that circPGR was exclusively localized in the cytosol of cells, which was consistent with the fact that most of exonic circRNAs were cytosolic (Figure 4G). *ACTIN* and U6 snoRNA were served as purity control for cytosolic and nuclear fractions, respectively (Figure 4G). The cytosolic localization of circPGR was further confirmed by RNA FISH by using a probe targeting the junction region (Figure 4H). To exclude the possibility that our probe might also target its parental gene *PGR*, we knocked down *PGR* and repeated the FISH experiment. Fluorescence intensity was not affected when *PGR* was knocked down, further supporting the specificity of our probe targeting circPGR (Figure S8E). Taken together, our data indicated that circPGR is an exonic circRNA stably localized in the cytosol of cells.

### Transcriptomics analysis reveals that circPGR regulates the expression of a cohort of cell cycle genes

**Figure 5:**
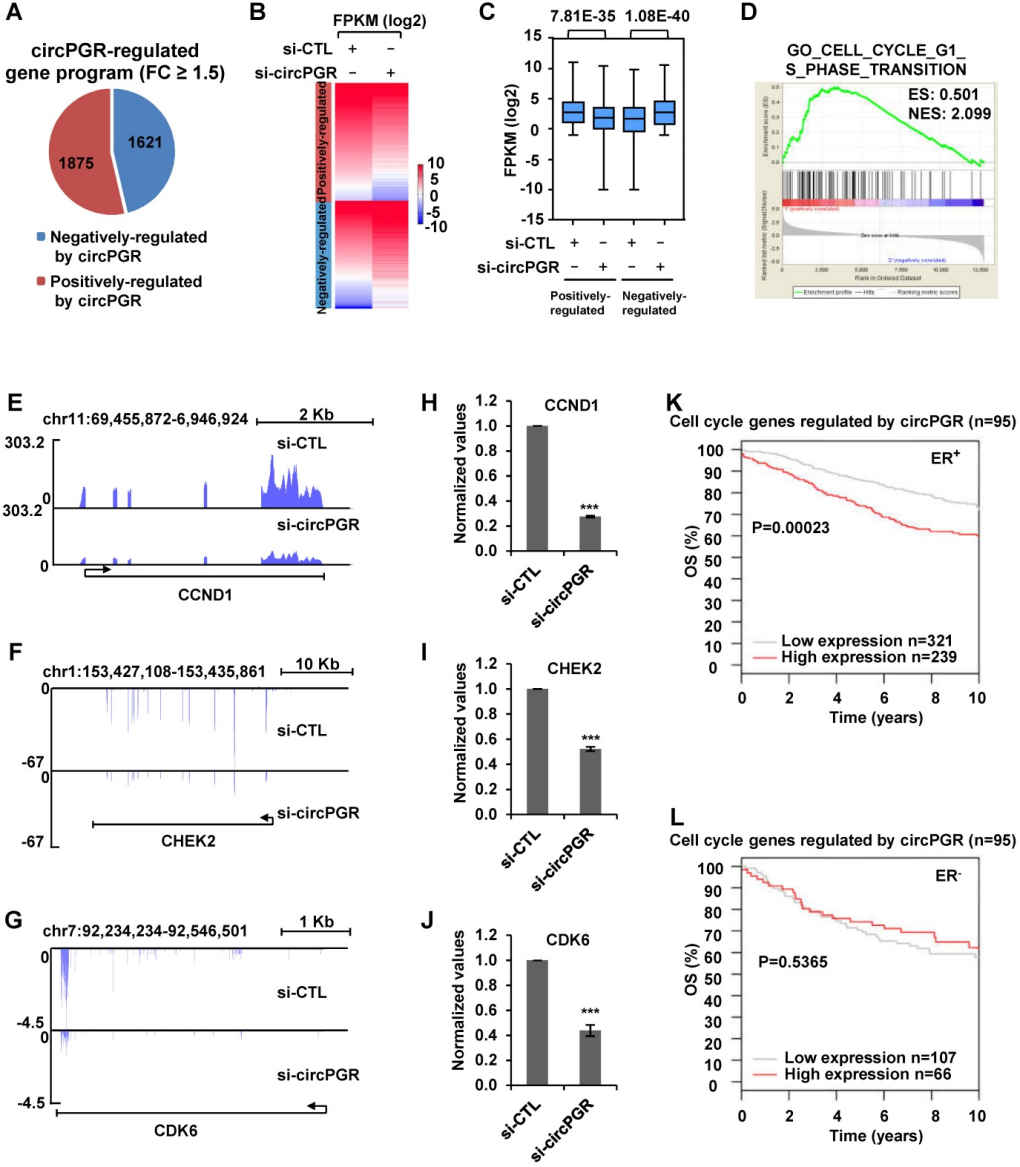
** Transcriptomics analysis reveals that circPGR regulates the expression of a cohort of cell cycle genes.** (**A**) MCF7 cells were transfected with control siRNA (si-CTL) or siRNA specific against circPGR (si-circPGR) followed by RNA-seq. Genes positively- and negatively- regulated by circPGR based on RNA-seq were shown by pie chart (fold change (FC) ≥ 1.5). (**B, C**) Heat map (B) and box plot (C) representation of the expression levels (FPKM (log2)) for genes positively- and negatively- regulated by circPGR based on RNA-seq as shown in (A). (**D**) The most enriched gene ontology (GO) term for genes positively-regulated by circPGR as revealed by GSEA gene ontology (GO) analysis. ES: enrichment score; NES: normalized enrichment score. (**E-G**) Genome browser views of RNA-seq as described in (A) for *CCND1* (E), *CHEK2* (F) and *CDK6* (G) were shown. (**H-J**) RNA samples as described in (A) were subjected to RT-qPCR analysis to examine the expression of *CCND1* (H), *CHEK2* (I) and *CDK6* (J) as indicated. Data shown were the relative fold change compared with control samples after normalization to actin (±s.e.m., ***P < 0.001). (**K, L**) Kaplan Meier survival analyses (OS, overall survival) for ER-positive (ER^+^) (K) and ER-negative (ER^-^) (L) breast cancer patients using circPGR-regulated cell cycle genes as input in GOBO.

We next sought to identify the target genes regulated by circPGR in order to gain insights into the molecular mechanisms underlying circPGR regulation of breast cancer cell growth. To this end, MCF7 cells were transfected with control siRNA or siRNA specifically targeting circPGR followed by RNA-seq analysis. It was found that there were 1,875 and 1,621 genes positively- and negatively-regulated by circPGR, respectively (Fold change (FC) ≥ 1.5) (Figure 5A and Table S2). The expression of these circPGR-regulated genes was shown by heat map and box plot (Figure 5B-C). GSEA 71 analysis for genes positively-regulated by circPGR revealed that Cell Cycle Regulation was the most enriched gene ontology (GO) term, which was consistent with the fact that circPGR was required for MCF7 cell cycle progression (Figure 5D). GO analysis by DAVID also revealed that the most enriched GO term was Cell Cycle Regulation (data not shown). CircPGR's effects on representative cell cycle genes from RNA-seq, such as *CCND1*,* CHEK2* and *CDK6*, were shown (Figure 5E-G), which were further confirmed by RT-qPCR analysis in both MCF7 and T47D cells (Figure 5H-J, Figure S9A-B). The downregulation of these cell cycle genes when knocking down of circPGR was not due to the downregulation of *PGR* (Figure S9C). To underscore the clinical relevance of genes positively-regulated by circPGR (n = 1,875), the high expression of these genes was found to be associated with poor prognosis in ER-positive, but not ER-negative breast cancer patients (Figure S9D-E). Particularly, association between the expression of those circPGR-regulated cell cycle genes and prognosis was even more significant (Figure 5K-L). Taken together, our RNA-seq analysis revealed that circPGR regulates the expression of a cohort of cell cycle genes, which might contribute to its function in controlling cell growth in ER-positive breast cancers.

### CircPGR acts as a ceRNA to sponge miR-301a-5p to regulate the expression of multiple cell cycle genes and cell growth

**Figure 6:**
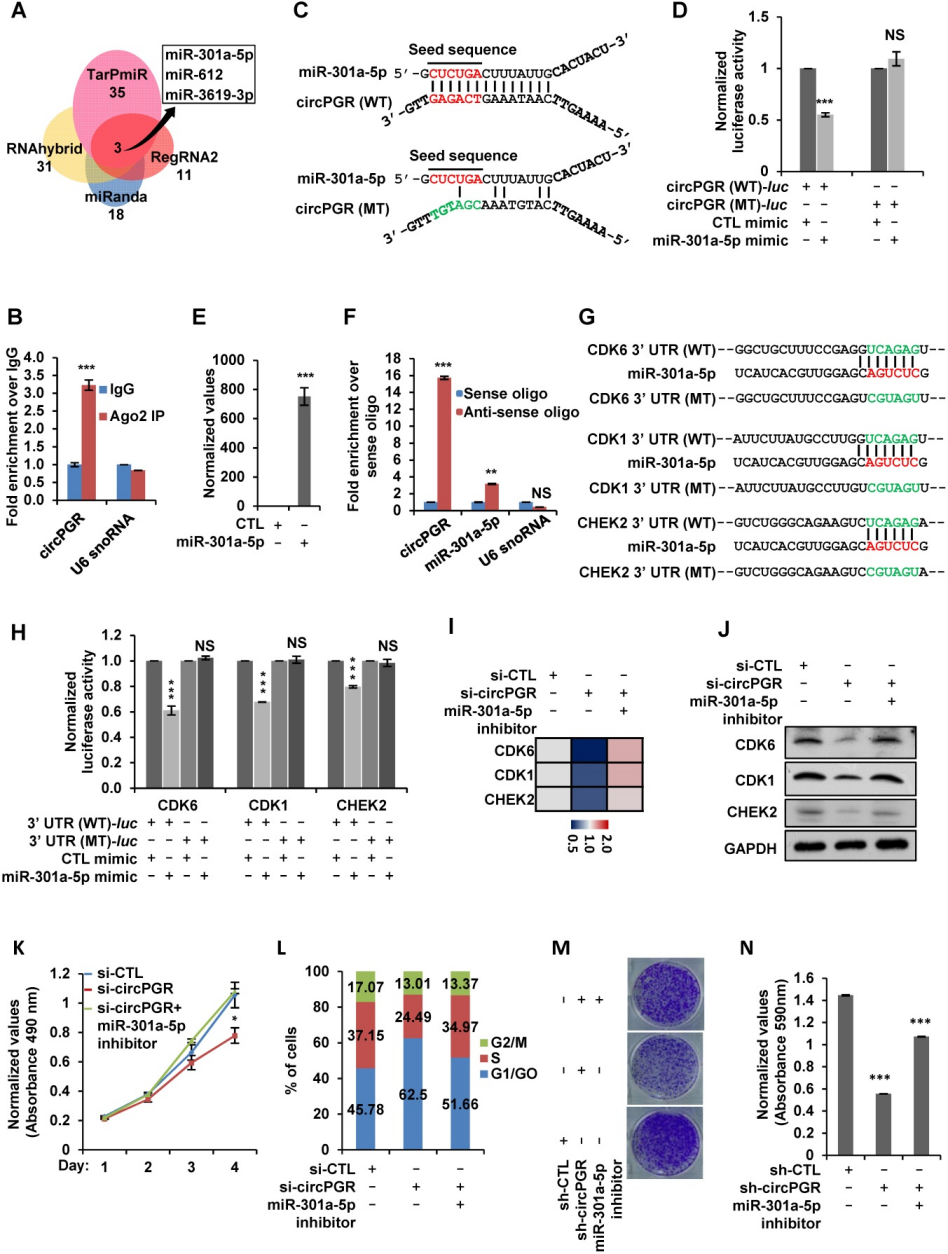
** CircPGR sponges miR-301a-5p to regulate the expression of multiple cell cycle genes and cell cycle progression.** (**A**) Four different miRNA prediction algorithms as indicted were used to predict potential miRNAs that can bind to circPGR, and the highly confident miRNAs predicted were then overlapped, resulting in three miRNAs, miR-301a-5p, miR-612 and miR-3619-3p. (**B**) RNA immunoprecipitation (RNA IP) was performed in MCF7 cells stably expressing 3 × Flag-tagged AGO2 by using control IgG or anti-Flag antibody followed by RT-qPCR to examine the binding of circPGR and U6 snoRNA (±s.e.m., ***P < 0.001). (**C**) Sequence match between miR-301a-5p and linear sequence of circPGR (circPGR (WT)) or circPGR mutant form with the potential miR-301a-5p binding site mutated (circPGR (MT)) was shown. Seed sequence was highlighted in red in miR-301a-5p. (**D**) Full length linear sequence of circPGR (circPGR (WT)) as well as its mutant form with the potential miR-301a-5p binding site mutated (circPGR (MT)) were cloned into a luciferase reporter vector, which were then transfected into HEK293T cells with control mimic or miR-301a-5p mimic followed by luciferase activity measurement (±s.e.m., ***P < 0.001, NS: non-significant). (**E**) The expression of miR-301a-5p mimic was detected by stem-loop RT-qPCR (±s.e.m., ***P < 0.001). (**F**) Oligonucleotide pull-down assay was performed by incubated MCF7 cell lysates with sense or anti-sense oligonucleotide targeting circPGR followed by RT-qPCR to detect the associated RNA as indicated (±s.e.m., **P < 0.01, ***P < 0.001, NS: non-significant). (**G**) Sequence match between miR-301a-5p and 3' untranslated region (UTR) of *CDK6*, *CDK1* or *CHEK2* (3' UTR (WT)) as well as the corresponding mutant form with the predicted miR-301a-5p binding site mutated (3' UTR (MT)) was shown. Seed sequence was highlighted in red in miR-301a-5p. (**H**) 3' UTR of *CDK6*, *CDK1* or *CHEK2* (3' UTR (WT)) as well as 3' UTR (MT) were cloned into a luciferase reporter vector, which were then transfected with control mimic (CTL mimic) or miR-301a-5p mimic into HEK293T cells followed by luciferase activity measurement. Data presented for miR-301a-5p mimic-transfected cells were fold induction over that of control mimic (±s.e.m., ***P < 0.001, NS: non-significant). (**I, J**) MCF7 cells transfected with control siRNA (si-CTL) or siRNA specifically targeting circPGR (si-circPGR) in the presence or absence of miR-301a-5p inhibitor were subjected to RT-qPCR (I) or immunoblotting (J) analysis to examine the expression of cell cycle genes as indicated. (**K, L**) MCF7 cells as described in (I) were subjected to cell proliferation assay (K) and FACS analysis (L) (±s.e.m., *P < 0.05). (**M**) MCF7 cells were infected with lenti-viral control shRNA (sh-CTL) or shRNA specifically targeting circPGR (sh-circPGR) followed by colony formation assay. (**N**) Quantification of the crystal violet dye as shown in (M) (± s.e.m., ***P < 0.001).

Next, we sought to examine the molecular mechanisms underlying circPGR regulation of cell cycle genes. As shown above, circPGR was found to be exclusively localized in the cytosol of cells, which prompted us to examine whether circPGR could function as a ceRNA to sponge miRNAs to regulate cell cycle genes. To this end, we utilized four different algorithms (RegRNA2.0 72, miRanda 73, RNAhybrid 74 and TarPmiR 75) to predict potential miRNAs that can bind to circPGR at high stringency, and then the highly confident miRNAs predicted were overlapped, resulting in three miRNAs, miR-301a-5p, miR-612 and miR-3619-3p (Figure 6A). To further understand how circPGR regulates cell cycle genes via its miRNA sponge function, we constructed circRNA (circPGR)-miRNA-mRNA (circPGR target genes) network, revealing that miR-301a-5p, miR-612 and miR-3619-3p were associated with different groups of circPGR target genes, with that miR-301a-5p targeted to many of the cell cycle genes circPGR regulated, such as *CDK6*, *CDK1* and *CHEK2* (Figure S10A). We therefore focused on studying ceRNA network containing circPGR, miR-301a-5p and circPGR-regulated cell cycle genes. We first examined whether circPGR can bind to miRNA by performing RNA immunoprecipitation for AGO2. It was found that circPGR, but not U6 snoRNA, was successfully pulled down by AGO2, suggesting that circPGR has the potential to bind with miRNAs (Figure 6B). We then examined whether miR-301a-5p can bind to circPGR by cloning the linear sequence of circPGR (circPGR (WT)) as well as its mutant form with the potential miR-301a-5p binding site mutated (circPGR (MT)) into a luciferase reporter vector, which were then transfected with control mimic or miR-301a-5p mimic into HEK293T cells followed by luciferase activity measurement. It was found that luciferase activity from vector containing circPGR (WT) (circPGR (WT)-*luc*), but not circPGR (MT) (circPGR (MT)-*luc*), was significantly inhibited by miR-301a-5p, suggesting that miR-301a-5p might bind to the predicted site in circPGR (Figure 6C-D). The expression of miR-301a-5p mimic was examined by RT-qPCR analysis (Figure 6E). To further validate the interaction between miR-301a-5p and circPGR, biotin-labelled sense or anti-sense oligonucleotides targeting circPGR was used for RNA pull-down, which revealed that circPGR as well as miR-301a-5p were significantly enriched by anti-sense oligonucleotides targeting circPGR, but not the sense oligonucleotides (Figure 6F). As expected, U6 snoRNA was not pulled down by either sense or anti-sense oligonucleotides (Figure 6F).

After knowing that miR-301a-5p binds to circPGR, we next tested whether miR-301a-5p regulates those circPGR-regulated cell cycle genes, such as *CDK1*, *CDK6* and *CHEK2*, as predicted in Figure S10A. To this end, we first cloned the 3' untranslated region (UTR) of *CDK6*, *CDK1* or *CHEK2* (3' UTR (WT)) as well as the corresponding mutant form with the predicted miR-301a-5p binding site mutated (3' UTR (MT)) into a luciferase reporter vector, which were then transfected with control mimic or miR-301a-5p mimic into HEK293T cells followed by luciferase activity measurement. It was found that luciferase activities from vector containing 3' UTR (WT), but not 3' UTR (MT) of *CDK6*, *CDK1* and *CHEK2* were significantly inhibited by miR-301a-5p, suggesting that miR-301a-5p binds to the predicted sites in the 3' UTR of these genes (Figure 6G-H). We also co-transfected circPGR with these luciferase reporter constructs, and found no evidence that circPGR could directly bind to the 3' UTRs of these genes (Figure S10B).

Having established miR-301a-5p's connection with circPGR and circPGR's target genes, such as *CDK6*, *CDK1* or *CHEK2*, we next sought to examine whether circPGR regulates these genes and cell growth was through sponging miR-301a-5p. Firstly, we transfected MCF7 cells with miR-301a-5p mimic or inhibitor, and found that transfection of miR-301a-5p mimic significantly attenuated, whereas miR-301a-5p inhibitor exhibited no effects on the proliferation and colony formation of MCF7 cells (Figure S10C-E). We then tested whether circPGR works through miR-301a-5p. To this end, MCF7 cells were transfected with control siRNA or siRNA specific targeting circPGR in the presence or absence of miR-301a-5p inhibitor. As expected, knockdown of circPGR led to a significant decrease of *CDK6*, *CDK1* and *CHEK2* expression at both mRNA and protein levels, which was rescued by miR-301a-5p inhibitor transfection, suggesting that circPGR regulation of these genes was dependent on miR-301a-5p (Figure 6I-J). Furthermore, circPGR regulation of cell cycle progression, cell growth and colony formation were also found to be dependent on miR-301a-5p as shown by cell proliferation assay (Figure 6K), FACS analysis (Figure 6L) and colony formation assay (Figure 6M-N), respectively. To support that circPGR could function as a ceRNA to sponge miR-301a-5p, the copy number of circPGR and miR-301a-5p was comparable in the presence of estrogen in MCF7 cells, which was 479 and 409 copies/cell, respectively (Figure S10F-G). Meanwhile, the copy number of *CDK1*, *CDK6* and *CHEK2* was measured to be 196, 9 and 8, respectively (Figure S10F-G). Taken together, circPGR acts as a ceRNA to sponge miR-301a-5p to regulate the expression of multiple cell cycle genes and breast cancer cell growth.

### CircPGR is highly expressed in ER-positive breast cancer cell lines and clinical breast cancer tissue samples, and anti-sense oligonucleotide (ASO)-targeting circPGR is effective in suppressing breast cancer cell growth

**Figure 7:**
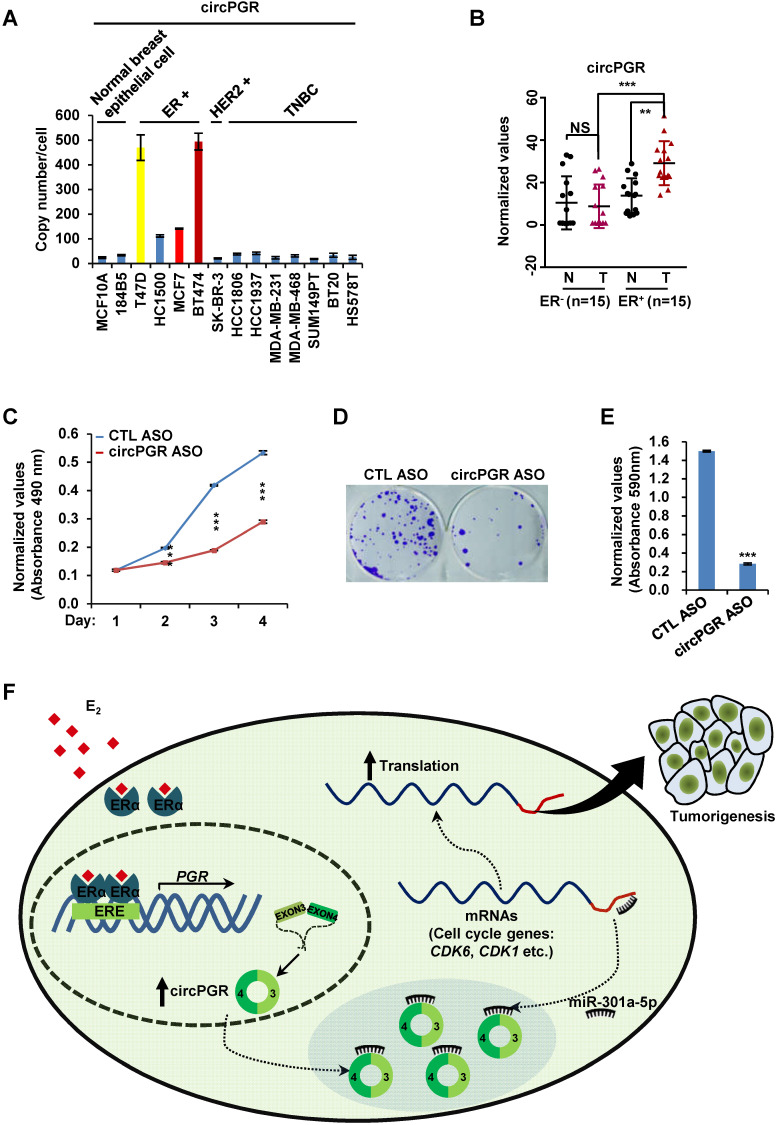
** CircPGR is highly expressed in ER-positive breast cancer cell lines and clinical breast cancer samples, and targeting circPGR is effective in suppressing breast cancer cell growth.** (**A**) RNA samples extracted from 14 different cell lines were subjected to RT-qPCR analysis to examine the expression of circPGR. Data shown was the absolute copy number. Normal breast epithelial cells: MCF10A and 184B5; ER-positive (ER^+^) cells: T47D, HC1500, MCF7 and BT474; HER2 (human epidermal growth factor receptor 2)-positive (HER2^+^) cells: SK-BR-3; TNBC (triple-negative breast cancer) cells: HCC1806, HCC1937, MDA-MB-231, MDA-MB-468, SUM149PT, BT20 and HS578T. (**B**) RNA samples extracted from 15 pairs of ER-negative breast tumor and adjacent normal tissues, and 15 pairs of ER-positive breast tumor and adjacent normal tissues were subjected to RT-qPCR analysis to examine the expression of circPGR. Data shown was normalized to actin (±s.e.m., **P < 0.01, ***P < 0.001, NS: non-significant). (**C, D**) MCF7 cells transfected with control ASO (CTL ASO) or ASO specifically targeting circPGR (circPGR ASO) were subjected to cell proliferation assay (C) or colony formation assay (D) (±s.e.m., ***P < 0.001). (**E**) Quantification of the crystal violet dye as shown in (D) (± s.e.m., ***P < 0.001). (**F**) A proposed model of circPGR function in ER-positive breast cancer: in the presence of estrogen, circPGR was co-induced with its parental gene, *PGR*, and later stably localized in the cytosol of cells, where it acted as a ceRNA to sponge miR-301a-5p to regulate the expression of multiple cell cycle genes. Alterations in estrogen signaling will result in the aberrant expression of circPGR and the release of cell cycle genes, such as *CDK6*, *CDK1* and *CHEK2*, from the repression by miR-301a-5p. Aberrant expression of these cell cycle genes will eventually drive breast tumorigenesis.

The functional importance of circPGR in ER-positive breast cancer cell growth suggested that it might be highly present in ER-positive breast cancer cells and targeting of circPGR might represent a new avenue to suppress the growth of ER-positive breast cancer cells. We first examined the expression of circPGR in breast epithelial cell lines as well as different subtypes of breast cancer cell lines, and found that circPGR was expressed at a much higher level in ER-positive cells (T47D, HC1500, MCF7 and BT474) compared to breast epithelial cells (MCF10A and 184B5), HER2 (human epidermal growth factor receptor 2)-positive cells (SK-BR-3) and TNBC (triple-negative breast cancer) cells (HCC1806, HCC1937, MDA-MB-231, MDA-MB-468, SUM149PT, BT20 and HS578T) (Figure 7A). Furthermore, circPGR was found to express at a significantly higher level in a cohort of ER-positive compared to ER-negative breast tumor tissues (Figure 7B). The levels of circPGR was significantly higher in ER-positive breast tumor tissues compared to that of adjacent normal tissues, and this type of difference between tumor and adjacent normal tissues was not observed in ER-negative breast tumors (Figure 7B). In consistent with the fact that circPGR regulates the expression of *CDK1*, *CDK6* and *CHEK2*, the expression of these genes was significantly higher in ER-positive breast tumor tissues compared to that of adjacent normal tissues (Figure S11A). The expression of miR-301a-5p was found to be similar (Figure S11B).

The high expression of circPGR in ER-positive breast cancer cells and its functional importance in regulating ER-positive breast cancer cell growth prompted us to examine whether targeting circPGR can suppress ER-positive breast cancer cell growth. MCF7 cells were transfected with control anti-sense oligonucleotide (ASO) or ASO specifically designed for circPGR followed by cell proliferation and colony formation assay. It was found that circPGR ASO significantly impaired cell proliferation as well as colony formation (Figure 7C-E). In conclusion, circPGR is highly expressed in ER-positive breast cancer cell lines and clinical breast tumor tissues, and ASO targeting circPGR is effective in suppressing breast cancer cell growth.

## Discussion

While estrogen/ER-induced transcriptional units, such as mRNAs, miRNAs, lncRNAs and eRNAs, have been shown to play a vital role in ER-positive breast cancer development, whether circRNAs can be induced by estrogen, and if so, what are the functional roles these circRNAs play in ER-positive breast cancers remain completely unknown. CircRNA-seq revealed that a large number of circRNAs were induced by estrogen in MCF7 cells, and exemplified by circPGR, estrogen-induced circRNAs might be functional in promoting breast cancer cell progression, which might represent a new class of therapeutic targets for ER-positive breast cancer treatment. As for circPGR, it was localized in the cytosol, where it worked as a ceRNA to sponge miR-301a-5p to regulate the expression of multiple cell cycle genes. Alterations in estrogen signaling will result in the aberrant expression of circPGR and the release of cell cycle genes from the repression by miR-301a-5p. Aberrant expression of these cell cycle genes will eventually drive breast cell growth (Figure 7F).

We sought out to uncover the estrogen-induced circRNA program by performing circRNA-seq in MCF7 cells, revealing a large number of circRNAs were induced by estrogen. Some of these estrogen-induced circRNAs were originated from estrogen-induced genes, and multiple circRNAs were often seen to be generated from the same gene. However, not all the circRNAs originated from the same estrogen-induced gene were estrogen-inducible, suggesting that different splicing machinery might be involved in the biogenesis of these circRNAs. On the other hand, the parental genes for many estrogen-induced circRNAs were not estrogen-inducible. Therefore, what triggers the expression of these circRNAs remains an interesting topic, and we propose that, similar as those circRNAs co-induced with their parental genes by estrogen, these circRNAs might still depend on ERα for its expression. Indeed, there were more than 30,000 ERα binding sites mapped in the genome when MCF7 cells were treated with estrogen 62, and only a few thousands of these ERα binding sites were linked to estrogen-induced gene transcription, while the functions of the rest remain unknown. One can envision that those ERα binding sites without assigned function might be involved in the induction of circRNAs that were independent on their parental genes. Therefore, a genome-wide integrative analysis of ERα binding sites and genomic locations where estrogen-inducible circRNAs were generated will provide insights into the biogenesis of these circRNAs.

The large number of estrogen-inducible circRNAs prompted us to examine the function of these circRNAs. Due to the fact that they were among the top most induced circRNAs by estrogen, the 6 circRNAs generated from PGR gene (circPGRs) were chosen as examples to test whether estrogen-induced circRNAs are functional in terms of regulating ER-positive breast cancer progression. Intriguingly, for the two circPGRs with designable siRNA, one, which we named as circPGR, was shown to be critical for ER-positive breast cancer progression including MCF7 and T47D. The functions of those four circRNAs without suitable siRNAs currently remain unclear.

The functional importance of circPGR in regulating ER-positive breast cancer cell growth inspired us to examine the underlying molecular mechanisms. Transcriptomics analysis revealed that the most prominent group of genes regulated by circPGR were related to cell cycle regulation, which was consistent with its critical role in promoting cell cycle progression and cell growth in ER-positive breast cancer. Similar as lncRNAs, circRNA can function either *in cis* or *in trans*. Several lines of evidence suggested that circPGR might function *in trans* to exert its function. Firstly, over-expression of circPGR was able to promote cell cycle progression and cell growth in both MCF7 and T47D cells. Secondly, circPGR was found to be exclusively localized in the cytosol of cells. The cytosolic-localized circPGR could regulate gene transcription by acting as a sponge for miRNA and/or scaffold for protein binding. In the current study, we focused on explore the possibility of circPGR functioning as a ceRNA to regulate gene expression. By doing ceRNA network analysis, three miRNAs, miR-301a-5p, miR-612 and miR-3619-3p, were predicted to be the intermediators between circPGR and its target genes. Particularly, miR-301a-5p bridged circPGR and multiple cell cycle genes, and miR-301a-5p later was proven to be involved in, at least partially, circPGR regulation of cell cycle gene expression, such as *CDK6*, *CDK1* and *CHEK2*, cell cycle progression and cell growth. A critical issue of the biological relevance of ceRNAs is whether they are functional under physiological conditions 76-78. To support that circPGR could function as a ceRNA to sponge miR-301a-5p, copy number analysis revealed that they were at the same order of magnitude. It should be noted that circPGR also regulated the expression of thousands of other genes, which are involved in sphingolipid metabolism, regulation of actin cytoskeleton, p53 signaling pathway and DNA replication, among others. It should also be noted that miR-301a-5p might also target to other estrogen-induced circRNAs, which might contribute to the observed ceRNA effects. CircPGR might also exert its functions through sponging other miRNAs, such as miR-612 and miR-3619-3p, as these two miRNAs were connected to a large of number of circPGR target genes with diverse functions.

Our data thereby revealed an estrogen-induced circRNA program, among which circPGR was shown to function as a ceRNA to regulate the expression of cell cycle-related genes and therefore cell cycle progression and cell growth in ER-positive breast cancer cells. The clinical relevance of circPGR was underscored by its high and specific expression in ER-positive breast tumor samples, suggesting that estrogen-induced circRNAs, similar as other estrogen-induced transcriptional products including mRNAs, miRNAs, lncRNAs and eRNAs, might serve as a new class of drug targets in ER-positive breast cancer. Therefore, exploring RNA therapies such as anti-sense oligonucleotide (ASO) and locked nucleic acid (LNA) targeting these circRNAs will provide a new avenue for treating ER-positive breast cancer.

## Materials and methods

### Clinical specimens and cell lines

Breast tumor tissues and matched adjacent normal tissues were obtained from patients who were diagnosed with breast cancer and who had undergone surgery at The Second Affiliated Hospital of Shantou University Medical College. Tissue samples were freshly frozen in dry ice and stored at -80 °C until RNA extraction. The study was approved by the Institutional Ethics Committee of the Second Affiliated Hospital of Shantou University Medical College. All research was performed in compliance with government policies and the Helsinki Declaration. Experiments were undertaken with the understanding and written consent of each subject.

Human breast cancer cell lines and human embryonic kidney cell line HEK293T were cultured in Dulbecco's modified Eagle medium (DMEM) (Biological Industries) or Roswell Park Memorial Institute 1640 (RPMI 1640) (Biological Industries). All medium was supplied with 10% fetal bovine serum (FBS) (Biological Industries) and 1% penicillin/streptomycin (Biological Industries) for cell culture. When treating cells with estrogen, phenol-free medium supplemented with 5% charcoal-treated FBS was used.

### Cloning procedures

ShRNAs targeting circPGR were cloned into lenti-viral pLKO.1 vector with AgeI and EcoRI restriction enzymes. (shRNA targeting sequence: AATCATTGCCAGGGCAGCACA (sh-circPGR#1); ATTGCCAGGGCAGCACAACTA (sh-circPGR#2)).

Full length circPGR was cloned in pCD2.1-ciR vector (Geneseed, Guangzhou) with KpnI and BamHI restriction enzymes for overexpression. Forward primer (F): 5'-GCCAAAGAATCCTGGGAGAT-3'; Reverse primer (R): 5'-CTGGCAATGATTTAGACCAC-3'. Linear sequence of circPGR (circPGR (WT)) as well as its mutant form with the potential miR-301a-5p binding site mutated (circPGR (MT)) were cloned into psiCHECK2 vector with NotI and XhoI restriction enzymes, which were named as circPGR (WT)-*luc* and circPGR (MT)-*luc*, respectively. Forward (F) primer: 5'-GGCAGCACAACTACTTAT-3'; Reverse (R) primer: 5'-CTGGCAATGATTTAGACC-3'. The primer used to mutate miR-301a-5p binding site was GTGCTCACAAACATCGTTTACATGAACTTTTTAA. 3' untranslated region (3' UTR) of *CDK6*,* CDK1* or *CHEK2* as well as the corresponding mutant form with the predicted miR-301a-5p binding site mutated were cloned into psiCHECK2 vector with NotI and XhoI restriction enzymes, which were named as 3' UTR (WT)-*luc* and 3' UTR (MT)-*luc*, respectively. CDK6-3' UTR (WT)-*luc*: forward primer: 5'-CAGTCTGAACCCCATTTG-3'; reverse primer: 5'- AGCTTAGCGCCTGAGAGA-3'. The primer used to mutate miR-301a-5p binding site was: TGCTTTCCGAGTCGTAGTTGCCCTGCTGCTGTCT. CDK1-3' UTR (WT)-*luc*: forward primer: 5'-CATCAGTTTCTTGCCATGT-3'; reverse primer: 5'- GAGCCTTTTTAGATGGCTG-3'. The primer used to mutate miR-301a-5p binding site was: AGTGAATTCTTATGCCTTGTCGTAGTTAATAACTGA. CHEK2-3' UTR (WT)-*luc*: forward primer: 5'-GCGCCTGAAGTTCTTGTT-3'; reverse primer: 5'- TGGGGTAGAGCTGTGGAT-3'. The primer used to mutate miR-301a-5p binding site was AAGTCTGGGCAGAAGTCCGTAGTAAAGCTCTGGACC.

Carboxyl (C)-terminal 3×Flag-tagged AGO2 was cloned into pCDH-CMV-puromycin vector with NotI and BamHI restriction enzymes: forward: 5'-ATGTACTCGGGAGCCGGC-3'; reverse: 5'-TCAAGCAAAGTACATGGT-3'.

### SiRNA, miRNA inhibitor, miRNA mimic and plasmids transfection, and lenti-virus packaging and infection

SiRNAs or anti-sense nucleotides (ASO) specifically targeting circPGR were purchased from RiboBio (siRNA targeting sequence: CCAGGGCAGCACAACUACU dTdT; ASO targeting sequence: GCCAGGGCAGCACAACUAC dTdT). miR-301a-5p mimic and inhibitor were also purchased from RiboBio. SiRNA transfections were performed using Lipofectamine 2000 (Invitrogen) according to the manufacturer's protocol. Plasmid transfections in HEK293T cells were performed using Polyethyleneimine (PEI, Polysciences) according to the manufacturer's protocol. Plasmids transfection in MCF7 cells were performed using Lipoplus reagent (Sagecreation) according to the manufacturer's protocol.

For Lenti-virus packaging: HEK293T cells were seeded in culture plates coated with poly-D-lysine (0.1% (w/v), Sigma, P7280) and transfected with lenti-viral vectors together with packaging vectors, pMDL, VSVG and REV, at a ratio of 10:5:3:2 using Polyethyleneimine (PEI, Polysciences) for 48 h according to the manufacturer's protocol. Virus were collected, filtered and added to MCF7 cells in the presence of 10 μg/mL polybrene (Sigma, H9268), followed by centrifugation for 30 min at 1,500 g at 37 °C. Medium was replaced 24 h later.

### RNA isolation and RT-qPCR

Total RNA was isolated using Trizol (Invitrogen) following the manufacturer's protocol. First-strand cDNA synthesis from total RNA was carried out using GoScript™ Reverse Transcription Mix (Promega, random primers), followed by quantitative PCR (qPCR) using AriaMx Real-Time PCR machine (Agilent Technologies). RNA samples from three biological repeats were pooled together for RT-qPCR analysis, and at least three technical repeats have been done for each pooled sample. Standard error of the mean is depicted. Sequence information for all primers used to check both gene expression and circPGR expression were presented in Supplementary Table 3 (Table S3).

### RNA sequencing (RNA-seq) and circRNA sequencing (circRNA-seq)

Total RNA was isolated using RNeasy Mini kit (Qiagen) following the manufacturer's protocol. DNase I in column digestion was included to ensure RNA quality. Two biological repeats were performed. For circRNA-seq, total RNA after DNase I treatment was digested with RNase R (Epicentre) at 37 °C for 1 h before RNA library preparation. RNA library preparation for both regular RNA-seq and circRNA-seq was performed by using NEBNext® Ultra™ Directional RNA Library Prep Kit for Illumina (E7420L). Paired-end sequencing was performed with Illumina HiSeq 3000 at RiboBio Co., Ltd. or Amogene Biotech Co., Ltd.

For regular RNA-seq, sequencing reads were aligned to hg19 reference genome by using TopHat (http://ccb.jhu.edu/software/tophat/index.shtml) 79. To determine circPGR-regulated gene program, Cuffdiff 80 was used to first quantify the expression of RefSeq annotated genes with the option -M (reads aligned to repetitive regions were masked) and -u (multiple aligned read are corrected using 'rescue method'). Coding genes with FPKM (fragments per kilobase per million mapped reads) larger than or equal to 0.5 in any one of the experimental conditions were included in our analysis. CircPGR-regulated gene program was determined by fold change (FC) of gene FPKM in si-CTL and si-circPGR-transfected samples (FC ≥ 1.5). FPKM of a gene was calculated as mapped reads on exons divided by exonic length and the total number of mapped reads.

For circRNA-seq analysis, sequencing reads were mapped to hg19 reference genome by using BWA MEM (-T 19), and circRNAs were predicted by using CIRI2.pl 81. To predict full length circRNAs, CIRI-full was used at default settings 81, 82. To visualize circRNAs, CIRI-vis was used at default settings 83.

Box plots were generated by R software and significance was determined using Student's t-test. Heat maps were visualized using Java TreeView or R software.

Gene ontology analysis (GO) was performed by using GSEA 71 or DAVID 84, and Kaplan Meier survival analysis was performed by using GOBO 85. Specifically, genes positively-regulated by circPGR or cell cycle genes positively-regulated by circPGR were submitted to GOBO, and “ER-positive” or “ER-negative” were selected for “Tumor selection”, “2 groups”, “10 years”, “overall survival” and “ER-status” were then selected. Results were exported in PDF format.

### Competitive endogenous RNA (CeRNA) network analysis

To construct ceRNA network, miRNAs that could bind to circPGR were first predicted by using four independent algorithms, miRanda (default settings) 73, RNAhybrid (minimal free energy, -e -23) 74, TarPmiR (probability of target site, -p 0.8) 75 and RegRNA2 72, based on miRBase. The miRNAs that were commonly predicted, miR-301a-5p, miR-612 and miR-3619-3p, were chosen for downstream analysis. mRNA targets that these three miRNAs could target to were predicted with Targetscan 86. To construct the ceRNA network for circPGR, only the mRNA targets that were shown to be regulated by circPGR were kept. The ceRNA network was constructed by Cytoscape 87.

### Cell Proliferation assay

Cell viability was measured by using a CellTiter 96 AQueous one solution cell proliferation assay kit (Promega) following the manufacturer's protocol. Briefly, MCF7 and T47D cells were transfected with si-circPGR or pCD2.1-circPGR and maintained in culture medium for different time points followed by cell proliferation assay. To measure cell viability, 20 µl of CellTiter 96 AQueous one solution reagent was added per 100 µl of culture medium, and the culture plates were incubated at 37 °C for 1 h in a humidified, 5% CO_2_ atmosphere incubator. The reaction was stopped by adding 25 µl of 10% SDS. Data was recorded at wavelength 490 nm using a Thermo Multiskan MK3 Microplate Reader.

### Fluorescence-activated cell sorting (FACS) analysis

Cells were trypsinized and then terminated by adding equal volume of medium, followed by centrifugation at 4 °C for 2 min (200 × g). Cell pellet was washed twice with cold PBS and fixed in 70% ethanol at 4 °C overnight. Ethanol was removed and cell pellet was washed twice with cold PBS. Cells were then stained with PI/Triton X-100 staining solution (0.1% (v/v) Triton X-100, 0.2 mg/mL DNase-free RNase A (Sigma), 0.02 mg/mL propidium iodide (PI, Roche)) at 37 °C for 15 min. DNA content was then measured by Attune NxT Flow Cytometer (Invitrogen) and about 10^5^ events were analyzed for each sample. Data were analyzed using ModFit LT (Verity Software House).

### Colony Formation Assay

Cells were seeded at the same density in 6-well plate and infected with lenti-viral shRNA, and medium was replaced 24 h later. Cells were then cultured in a humidified, 5% CO_2_ atmosphere incubator at 37 °C for 2-3 weeks until colonies developed. The cells were fixed in fixative solution (acetic acid: methanol =1:3) for 15 min and stained with 0.1% crystal violet for 15 min. For quantification, the crystal violet dye was released into 10% acetic acid and data was recorded at wavelength 590 nm.

### Wound healing assay

SiRNA-transfected cells were re-seeded at confluence in 6-well plates, and wounds were performed with a P200 pipette tip. After removing cellular debris by washing cells with PBS, three images of each well were taken. The wounded area was measured by using image J and recorded as A0. The cells were then allowed to migrate into the wounded area, and three images were taken and the wounded area was measured again 36 or 48 h later and recorded as A1. Cell migration was presented as wound closure (%) = (wounded area (A0-A1)/wounded area A0) × 100%.

### Trans-well assay

SiRNA-transfected cells were re-seeded on the top compartment of trans-well Boyden chambers (8 μm, Corning, USA) in serum-free media, and then allowed to migrate to the lower compartment contained complete media with 10% FBS (fetal bovine serum) in a humidified, 5% CO_2_ atmosphere incubator at 37 °C for 36 h. Cells that did not migrate into the lower compartment were wiped away with a cotton swab. The inserts were fixed with fixative solution (acetic acid: methanol = 1:3) for 15 min and stained with 0.1% crystal violet for 10 min. After washing with PBS extensively, migrated cells were photographed and quantified using Image J. Three images of each well were taken and representative images were shown.

### Tumor xenograft assay

Three groups (5 mice/group) of female BALB/C nude mice (age 4-6 weeks) were subcutaneously implanted with 1×10^7^ of sh-CTL, sh-circPGR#1, sh-circPGR#2-infected MCF7 cells suspended in PBS. Each nude mouse was brushed with estrogen (E_2_, 10^-2^ M) every 3 days for the duration of the experiments to sustain xenograft tumor growth. All mice were euthanized 20 days after subcutaneous injection. Tumors were then excised, photographed and weighted. Animals were housed in the Animal Facility at Xiamen University under pathogen-free conditions, following the protocol approved by the Xiamen Animal Care and Use Committee.

### RNA fluorescence *in situ* hybridization (RNA-FISH)

MCF7 cells seeded on cover glass were transfected with siRNAs for 48 h before fixing with fixation buffer (4% formaldehyde, 10% acetic acid, 1 × PBS) for 10 min. Cells were then permeabilized in 70% of ethanol overnight and rehydrated in 2 × SSC buffer (300 mM NaCl, 30 mM sodium citrate (pH7.0)) with 50% formamide. Hybridization was carried out in the presence of 30 ng of probe (Sangon Biotech, biotin-aaaGTAGTTGTGCTGCCCTGGCAATGATTTAGAC) at 37 °C overnight. Biotin-labeled probes were incubated with Streptavidin-Cy3™ (Sigma Aldrich, S6402) in 2 × SSC buffer with 8% formamide, 2 mM vanadyl-ribonucleoside complex, 0.2% RNase-free BSA at 37 °C for 1 h in dark. Nuclei were counterstained with DAPI (0.1 µg/mL) and then washed twice with 2 × SSC with 8% formamide at room temperature (RT) for 15 min. Three images of each cover glass were taken with Carl Zeiss laser confocal microscope, and representative images were shown.

### Cellular fractionation

Cellular fractionation was performed as described previously 88. Briefly, cells were washed with ice-cold PBS, collected, spun down and re-suspended in ice-cold buffer I (10 mM Hepes, pH 8.0, 1.5 mM MgCl_2_, 10 mM KCl, 1 mM DTT) supplemented with protease inhibitor cocktail, followed by incubation for 15 min on ice to allow cells to swell. Igepal-CA630 was then added at a final concentration of 1% (use 10% stock solution) followed by vortexing for 10 s. Nuclei were collected by centrifuging 2∼3 min at maximum speed (∼21,100 × g). The resultant supernatant was cytosolic fraction. Nuclei were then lysed in ice cold buffer II (20 mM Hepes, pH 8.0, 1.5 mM MgCl2, 25% (v/v) glycerol, 420 mM NaCl, 0.2 mM EDTA, 1 mM DTT) supplemented with protease inhibitor cocktail followed by vigorous rotation at 4 °C for 30 min and centrifugation 15 min at maximum speed. The resultant supernatant was nuclear fraction. Both cytosolic and nuclear RNAs were extracted by Phenol-Chloroform-Isoamyl Alcohol mixture (Sigma, 77618) followed by RT-qPCR analysis.

### RNase R digestion

Total RNAs were isolated by TRIzol which were treated with RNase R (Epicentre) (10 units/µg RNA) in RNase R buffer supplemented with murine Ribonuclease Inhibitor (New England Biolabs) at 37 °C for 1 h. First-strand cDNA synthesis from RNase R-treated RNA was carried out using GoScript™ Reverse Transcription Mix (Promega, random primers), followed by quantitative PCR (qPCR) using AriaMx Real-Time PCR machine (Agilent Technologies).

### RNA immunoprecipitation (RNA-IP)

RNA-IP was performed as described previously 89. Briefly, cells stably expressing 3 × Flag-tagged AGO2 were lysed in polysome lysis buffer (100 mM KCl, 5 mM MgCl_2_, 10 mM HEPES (pH 7.0), 0.5% NP-40, 1 mM DTT, 100 U/ml RNasin RNase inhibitor (Promega, N2511), 2 mM vanadyl ribonucleoside complexes solution (Sigma, 94742), 25 μl/ml protease inhibitor cocktail (Sigma, P8340)), which were then subjected to IP by using M2 agarose (Sigma, F1804) followed by washing with polysome lysis buffer four times and polysome lysis buffer plus 1 M urea four times. RNAs was released by adding 150 μl of polysome lysis buffer with 0.1% SDS and 45 μg proteinase K (Ambion, AM2548) and incubated at 50 °C for 30 min. RNA extracted with phenol-chloroform-isoamyl alcohol mixture (Sigma, 77618) was recovered by adding 2 μl GlycoBlue (15 mg/ml, Ambion, AM9516), 36 μl 3 M sodium acetate and 750 μl ethanol followed by incubation at -20 °C for overnight. Precipitated RNAs were washed with 70% ethanol, air dried, and re-suspended in RNase free water followed by DNase I (Promega, M6101) treatment to remove genomic DNA. The resultant RNAs were subjected to RT-qPCR analysis.

### Anti-sense or sense oligonucleotide pull-down assay

Oligonucleotide pull-down was carried out as described previously 90. MCF7 cells were lysed in polysome extraction buffer (PEB, 20 mM Tris-HCl (pH 7.5), 100 mM KCl, 5 mM MgCl2 and 0.5% NP-40) supplemented with protease inhibitor cocktail and RNase inhibitor for 10 min on ice and the supernatant was collected by centrifugation (15,000 × g, 10 min, 4 °C), which were then incubated with 100 pmol of biotin-labeled sense oligonucleotide (biotin-aaaGGAACCTGAATGTGCACCTTCGGGCTGTCTCC) or an anti-sense oligonucleotide complementary to the junction sequence of circPGR (biotin-aaaGTAGTTGTGCTGCCCTGGCAATGATTTAGAC) in 1 × TENT buffer (10 mM Tris-HCl (pH 8.0), 1 mM EDTA (pH 8.0), 250 mM NaCl, 0.5% Triton X-100 (v/v)) containing protease inhibitor cocktail and RNase inhibitor at 25 °C for 1 h with rotation. Streptavidin-coupled Dynabeads (Invitrogen M280) were washed with 1 × TENT buffer and then added at 25 °C for 30 min with rotation. After washing the beads three times with ice cold 1×TENT buffer, RNA was isolated using TRIzol and circPGR-associated microRNAs in the pull down were detected by RT-qPCR analysis.

### Immunoblotting

Immunoblotting was performed following the protocol described previously 70. Anti-CHEK2 (A-12) (sc-5278), anti-GAPDH (0411) (sc-47724) and anti-CDK1 (17) (sc-54) antibodies were purchased from Santa Cruz Biotechnology; anti-CDK6 (19117-1-AP) antibody was purchased from Proteintech.

### Copy number analysis

Copy number analysis was performed following the protocol reported previously with minor modifications 91. Briefly, MCF7 cells cultured in stripping medium for three days were treated with estrogen (10^-7^ M) for 6 h, and 1.5 × 10^6^ cells were then used for copy number analysis. Absolute quantification was performed using 2-fold serial dilutions of the reference standard, and Ct value from qPCR analysis versus the dilution factor was plotted, fitting the data to a straight line. The standard curve was used for extrapolating the number of molecules of interest.

### Statistical analysis

The comparison of two groups or data points was performed by the two-tailed t-test. Multiple comparisons were analyzed by two-way analysis of variance (ANOVA). Survival curves were constructed according to the GOBO method and compared using the log-rank (Mantel-Cox) test. P ≤ 0.05 were considered statistically significant. Results from xenograft experiments and clinical breast samples were analyzed by GraphPad Prism 7.

## Supplementary Material

Supplementary figures.

Supplementary table S1.

Supplementary table S2.

Supplementary table S3.
