# Supplementary material for: Estrogen-induced circRNA, circPGR, functions as a ceRNA to promote estrogen receptor-positive breast cancer cell growth by regulating cell cycle-related genes
Source: Theranostics. 2021 Jan 1;11(4):1732–52. doi: 10.7150/thno.45302 (PMC7778588; doi:10.7150/thno.45302)
Supplement: Supplementary file 1 — Supplementary figures. [file thnov11p1732s1.pdf]

Supplementary information

Supplementary figure legends

Figure S1. A large number of circRNAs are detected from circRNA-seq in MCF7 cells.

(A) Genome browser views of RNA-seq data for selected genes as indicated were shown. Blue: control (CTL); Red: estrogen (E_2).

(B) Flowchart to predict circRNAs by using CIRI2.

(C) The correlation of junction reads between circRNAs identified in two biological repeats.

(D) Statistics of circRNAs with different number of junction reads predicted by CIRI2 were shown.

(E, F) Genomic location and schematic representation of TTN (E) and NEAT1 (F) gene (top) and the circRNA produced from the same gene region (bottom). CircRNA was highlighted in light blue.

(G-J) Four randomly selected circRNAs predicted by CIRI2 were PCR amplified by using divergent primer sets specifically targeting the junction regions followed by Sanger sequencing. Sequence flanking junction regions was shown. Junction sites were highlighted in light blue. Sanger sequencing histogram was shown at the bottom.

Figure S2. Comparison of CircRNAs predicted by different algorithms.

(A-C) Comparison of circRNAs predicted by CIRI2, find_circ and CIRCexplorer2 under cutoff of junction reads of 2 (A), 5 (B) or 10 (C).

(D, H) Correlation between the number of exons a gene has and circRNAs originated from that gene based on circRNAs predicted by CIRCexplorer2 (D) or find_circ (H).

(E, I) Correlation between the expression of parental gene (FPKM) and circRNA originated from the same gene region (average junction reads/gene) based on circRNAs predicted by CIRCexplorer2 (E) or find_circ (I).

(F, J) Box plot showing the length of randomly selected introns or introns flanking, upstream and downstream, circRNA-producing regions based on circRNAs predicted by CIRCexplorer2 (F) or find_circ (J).

(G, K) Percentage of randomly selected introns or introns flanking circRNA-producing regions containing inverted repeated Alu pairs based on circRNAs predicted by CIRCexplorer2 (G) or find_circ (K).

Figure S3. A large number of circRNAs are induced by estrogen.

(A, B) Venn diagram showing overlapping between circRNAs predicted in both control (CTL) and estrogen (E_2)-treated conditions (junction reads ≥ 5 (A) or 10 (B), FC ≥ 2).

(C) Genomic location, parental gene, strand information, junction reads in both control (CTL) and estrogen (E_2)-treated conditions, number of cycles detected from RT-qPCR analysis, and fold induction deduced from RT-qPCR analysis of circRNAs shown in Fig. 2B.

Figure S4. PGR has no significant impact on MCF7 cell growth.

(A) MCF7 cells transfected with control siRNA (si-CTL) or siRNA specifically targeting circPGR (si-circPGR) were subjected to RNA extraction and RT-qPCR analysis to examine the expression of *PGR* (± s.e.m., ***P < 0.01).

(B) MCF7 cells transfected with control siRNA (siCTL) or two independent siRNAs specifically targeting *PGR* (siPGR#1 and siPGR#2) were subjected to RNA extraction and RT-qPCR analysis to examine the expression of *PGR* (± s.e.m., ***P < 0.01).

(C) MCF7 cells transfected with control siRNA (siCTL) or two independent siRNAs specifically targeting *PGR* (siPGR#1 and siPGR#2) were cultured in stripping medium and treated with or without estrogen (E_2, 10^{-7} M) for duration as indicated, followed by cell proliferation assay (NS, non-significant).

Figure S5. circPGR promotes growth, migration and invasion in MCF7 cells.

(A) MCF7 cells were transfected with control vector (circCTL) or vector expressing circPGR followed by RT-qPCR to examine the expression of circPGR (± s.e.m., ***P < 0.001).

(B, C, D, F, H) MCF7 cells as described in (A) were subjected to cell proliferation assay (B), FACS analysis (C), colony formation assay (D), wound healing assay (F), and transwell assay (H) (± s.e.m., *P < 0.05).

(E, G, I) Quantification of the crystal violet dye (D), wound closure (F) and number of colonies (H) as shown in (E), (G) and (I), respectively (± s.e.m., *P < 0.05, ***P < 0.001).

Figure S6. Knockdown of circPGR inhibits growth, migration and invasion in T47D cells.

(A, B, E, G) T47D cells were transfected with control siRNA (siCTL) or siRNA specifically targeting circPGR (si-circPGR#1) followed by cell proliferation assay (A), FACS analysis (B), wound healing assay (E), and transwell assay (G) (± s.e.m., *P < 0.05, **P < 0.01).

(C) T47D cells were infected with lenti-viral control shRNA (sh-CTL) or shRNA specifically targeting circPGR (sh-circPGR) followed by colony formation assay (± s.e.m., ***P < 0.001).

(D, F, H) Quantification of the crystal violet dye (D), wound closure (F) and number of colonies (H) as shown in (C), (E) and (G), respectively (± s.e.m., *P < 0.05, ***P < 0.001).

Figure S7. circPGR promotes growth, migration and invasion in T47D cells.

(A, B, C, E, G) T47D cells were transfected with control vector (circCTL) or vector expressing circPGR followed by cell proliferation assay (A), FACS analysis (B), colony formation assay (C), wound healing assay (E), and transwell assay (G) (± s.e.m., **P < 0.01, ***P < 0.001).

(D, F, H) Quantification of the crystal violet dye (D), wound closure (F) and number of colonies (H) as shown in (C), (E) and (G), respectively (± s.e.m., *P < 0.05, **P < 0.01, ***P < 0.001).

Figure S8. CircPGR is stably localized in the cytosol of cells.

(A) T47D cells cultured in stripping medium and treated with or without estrogen (E_2, 10^{-7} M) for duration as indicated were subjected to RNA extraction, reverse transcription and standard PCR by using primer sets specifically targeting circPGR or *PGR*. The resultant PCR products were separated by DNA electrophoresis. Actin was served as a loading control. DNA marker (M) was shown as indicated. bp: base pair.

(B) MCF7 cells were cultured in stripping medium and treated with or without estrogen (E_2, 10^{-7} M, 1 h) followed by ERα ChIP-seq analysis, and UCSC genome browser views of ERα binding on *PGR* gene region as well as distal enhancer (E) were shown. N.C: negative control region; TSS: transcription start site; Blue: control (CTL); Red: estrogen (E_2).

(C) MCF7 cells were cultured in stripping medium and treated with or without estrogen (E_2, 10^{-7} M, 1 h) followed by ChIP-qPCR analysis to examine the binding of ERα. Data shown was the percentage of input (± s.e.m., ***P < 0.001, NS: non-significant).

(D) MCF7 cells were cultured in stripping medium and pre-treated with or without ICI 182,780 (ICI, 10^{-7} M) for 12 h before estrogen (E_2, 10^{-7} M) treatment for another 6 h followed by RNA extraction and RT-qPCR analysis to examine the expression of circPGR. Data shown was the relative fold change compared to control samples (CTL) after normalization to actin (± s.e.m, ***P < 0.001).

(E) CircPGR RNA-FISH analysis was performed in MCF7 cells transfected with control siRNA (siCTL) or two independent siRNA targeting *PGR* (siPGR#1 and siPGR#2) and treated with estrogen (E_2, 10^{-7} M, 6 h). Red: circPGR; Blue: DAPI.

Figure S9. circPGR regulates the expression of a cohort of cell cycle genes in T47D cells.

(A, B) T47D cells transfected with control siRNA (si-CTL) or two independent siRNAs specific against circPGR (si-circPGR#1 and si-circPGR#2) were subjected to RT-qPCR analysis to

examine the expression of circPGR (A), *CCND1*, *CHEK2* and *CDK6* (B) as indicated. Data shown were the relative fold change compared with control samples after normalization to actin (±s.e.m., ***P < 0.001).

(C) T47D cells transfected with control siRNA (siCTL) or two independent siRNAs specific against *PGR* (siPGR#1 and siPGR#2) were subjected to RT-qPCR analysis to examine the expression of *CCND1*, *CHEK2* and *CDK6* as indicated. Data shown were the relative fold change compared with control samples after normalization to actin (±s.e.m., **P < 0.01, NS: non-significant).

(D, E) Kaplan Meier survival analyses (OS, overall survival) for ER-positive (ER^+) (D) and ER-negative (ER^-) (E) breast cancer patients using genes positively-regulated by circPGR as input in GOBO.

Figure S10. CircPGR acts as a ceRNA to promote cell cycle progression.

(A) CeRNA network analysis revealed that circPGR sponge three miRNAs with high confidence, miR-301a-5p, miR-3619-3p and miR-812, to regulate its target genes.

(B) 3' UTR of *CDK6*, *CDK1* or *CHEK2* (3' UTR (WT)) as well as 3' UTR (MT) were cloned into a luciferase reporter vector, which were then transfected into HEK293T cells with control vector (circCTL) or vector expressing circPGR followed by luciferase activity measurement (NS: non-significant).

(C, D) MCF7 cells were transfected with control mimic (CTL mimic), miR-301a-5p inhibitor or miR-301a-5p mimic followed by cell proliferation assay (C) and colony formation assay (D). (±s.e.m., *P < 0.05, NS: non-significant).

(E) Quantification of the crystal violet dye as shown in (D) (± s.e.m., ***P < 0.001, NS: non-significant).

(F) The standard curves for copy number analysis of molecules as indicated were shown. Two-fold serial dilutions of reference standard was first performed, and Ct value from qPCR analysis versus the dilution factor was plotted, fitting the data to a straight line.

(G) MCF7 cells cultured in stripping medium for three days were treated with or without estrogen (10^{-7} M) for 6 h, and 1.5 x 10^{6} cells were then used for copy number analysis based on the standard curves as shown in (F).

Figure S11. CircPGR's target genes are highly expressed in clinical breast cancer samples. (A, B) RNA samples extracted from 15 pairs of ER-negative breast tumor and adjacent normal tissues, and 15 pairs of ER-positive breast tumor and adjacent normal tissues were subjected to RT-qPCR analysis to examine the expression of *CDK1*, *CDK6* and *CHEK2* (A) and miR-301a-5p (B). Data shown was normalized to actin (±s.e.m., *P < 0.05, **P < 0.01, NS: non-significant).

Supplementary table legends

Table S1. List of circRNAs detected from circRNA-seq in both control (CTL) and estrogen (E_2)-treated conditions in MCF7 cells.

Table S2. List of genes positively- and negatively-regulated by circPGR in MCF7 cells.

Table S3. Sequence information for all qPCR primers used in the current study. Sequence information of qPCR primers designed to detect the expression of mRNAs or circRNAs were shown. F: forward; R: reverse.

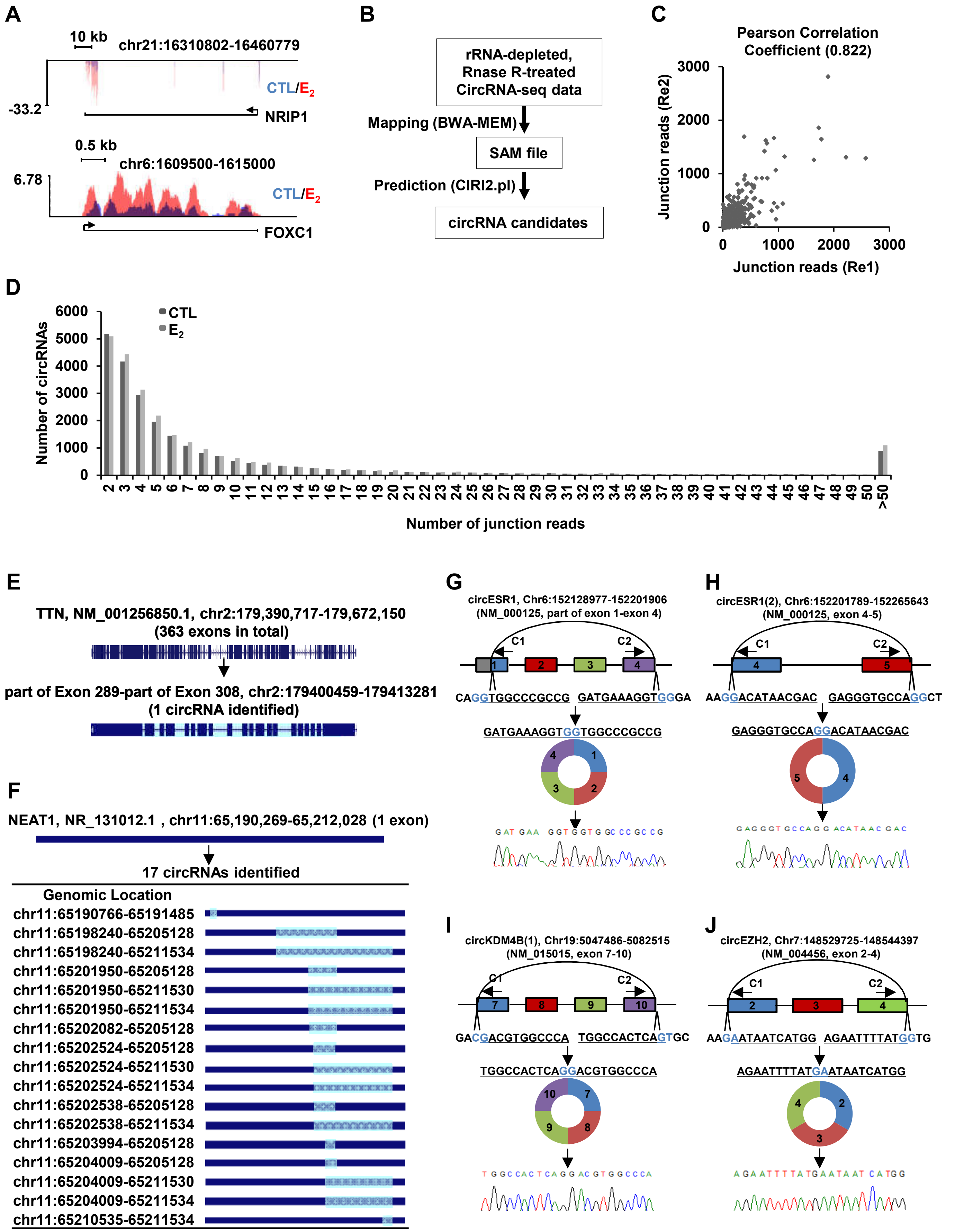
A
10 kb
chr21:16310802-16460779
-33.2
CTL/E2
NRIP1
0.5 kb
chr6:1609500-1615000
6.78
CTL/E2
FOXC1
B
rRNA-depleted, Rnase R-treated CircRNA-seq data
Mapping (BWA-MEM)
SAM file
Prediction (CIRI2.pl)
circRNA candidates
C
Pearson Correlation Coefficient (0.822)
Junction reads (Re2)
Junction reads (Re1)
D
CTL
E2
Number of circRNAs
Number of junction reads
E
TTN, NM_001256850.1, chr2:179,390,717-179,672,150
(363 exons in total)
part of Exon 289-part of Exon 308, chr2:179400459-179413281
(1 circRNA identified)
F
NEAT1, NR_131012.1 , chr11:65,190,269-65,212,028 (1 exon)
17 circRNAs identified
Genomic Location
chr11:65190766-65191485
chr11:65198240-65205128
chr11:65198240-65211534
chr11:65201950-65205128
chr11:65201950-65211530
chr11:65201950-65211534
chr11:65202082-65205128
chr11:65202524-65205128
chr11:65202524-65211530
chr11:65202524-65211534
chr11:65202538-65205128
chr11:65202538-65211534
chr11:65203994-65205128
chr11:65204009-65205128
chr11:65204009-65211530
chr11:65204009-65211534
chr11:65210535-65211534
G
circESR1, Chr6:152128977-152201906
(NM_000125, part of exon 1-exon 4)
C1
C2
CAGGTGGCCCGCCG GATGAAAGGTGGGA
GATGAAAGGTGGTGGCCCGCCG
H
circESR1(2), Chr6:152201789-152265643
(NM_000125, exon 4-5)
C1
C2
AAGGACATAACGAC GAGGGTGCCAGGCT
GAGGGTGCCAGGACATAACGAC
I
circKDM4B(1), Chr19:5047486-5082515
(NM_015015, exon 7-10)
C1
C2
GACGACGTGGCCCA TGGCCACTCAGTGC
TGGCCACTCAGGACGTGGCCCA
J
circEZH2, Chr7:148529725-148544397
(NM_004456, exon 2-4)
C1
C2
AAGAATAATCATGG AGAATTTTATGGTG
AGAATTTTATGAATAATCATGG

Figure S1

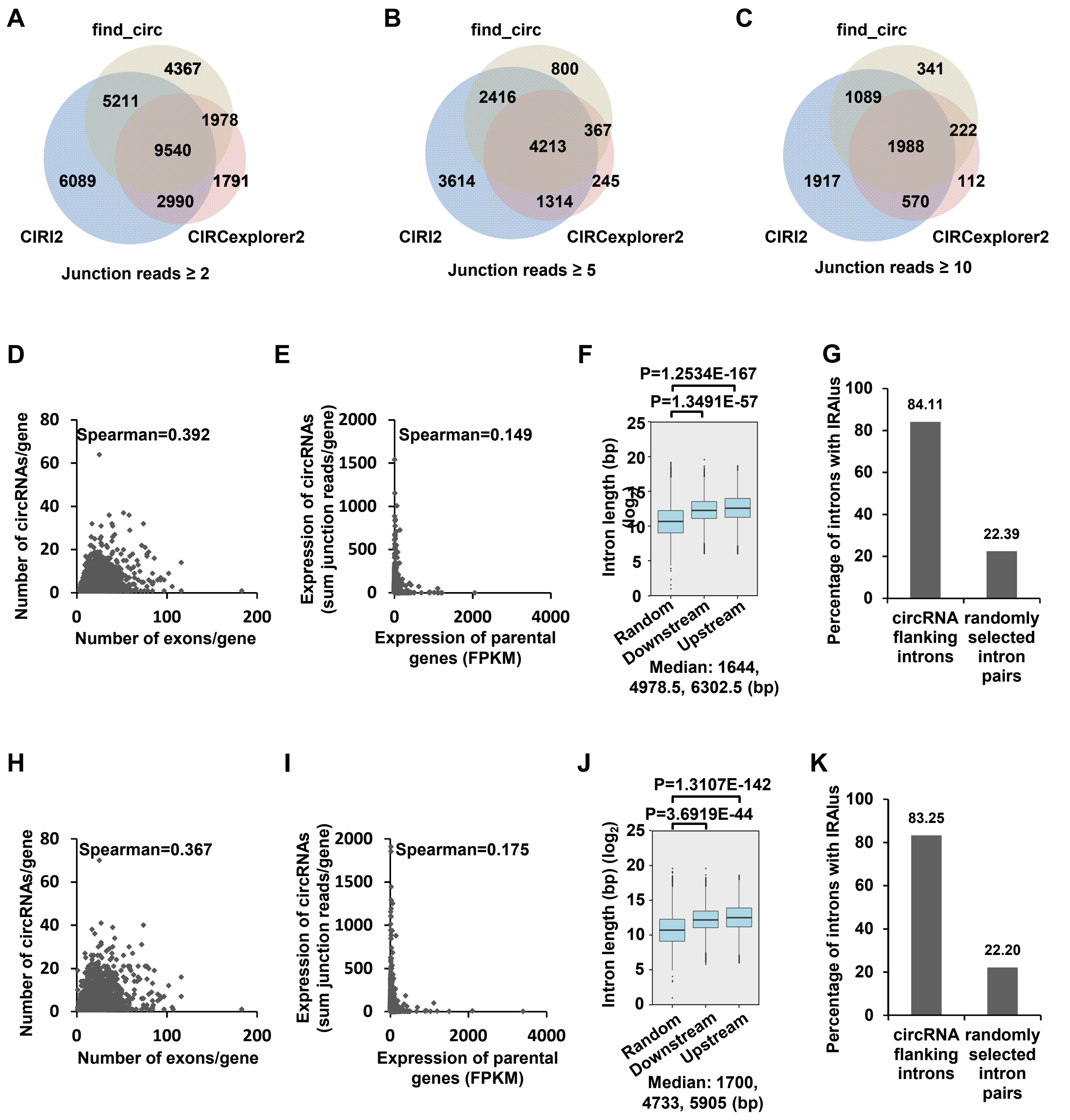

A
find_circ
4367
5211
1978
9540
6089
1791
2990
CIRI2
CIRCexplorer2
Junction reads ≥ 2
B
find_circ
800
2416
367
4213
3614
245
1314
CIRI2
CIRCexplorer2
Junction reads ≥ 5
C
find_circ
341
1089
222
1988
1917
112
570
CIRI2
CIRCexplorer2
Junction reads ≥ 10
D
Spearman=0.392
Number of circRNAs/gene
Number of exons/gene
E
Spearman=0.149
Expression of circRNAs (sum junction reads/gene)
Expression of parental genes (FPKM)
F
P=1.2534E-167
P=1.3491E-57
Intron length (bp) (log2)
Random
Downstream
Upstream
Median: 1644, 4978.5, 6302.5 (bp)
G
Percentage of introns with IRAlus
84.11
22.39
circRNA flanking introns
randomly selected intron pairs
H
Spearman=0.367
Number of circRNAs/gene
Number of exons/gene
I
Spearman=0.175
Expression of circRNAs (sum junction reads/gene)
Expression of parental genes (FPKM)
J
P=1.3107E-142
P=3.6919E-44
Intron length (bp) (log2)
Random
Downstream
Upstream
Median: 1700, 4733, 5905 (bp)
K
Percentage of introns with IRAlus
83.25
22.20
circRNA flanking introns
randomly selected intron pairs

Figure S2

A

circRNAs with junction reads ≥ 5

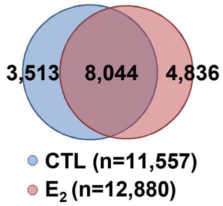

B

circRNAs with junction reads ≥ 10

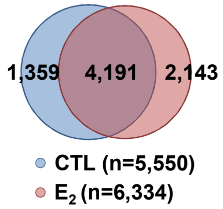

C

CircRNA	Chr	Start	End	Parental gene	Strand	CircRNA type	Junction reads (CTL)	Junction reads (E_2)	Ct value (CTL, mean)	Ct value (E_2, mean)	Fold change (E_2/CTL)
circPGR(6)	chr11	100933178	100996889	PGR	-	exon	0	23	24.22	21.93	4.636957
circFMN1(1)	chr15	33180373	33194241	FMN1	-	exon	8	12	24.41	22.13	3.854164
circCA12(2)	chr15	63637676	63638908	CA12	-	exon	0	4	22.02	20.31	3.204261
circLETMD1	chr12	51442817	51451911	LETMD1	+	exon	5	26	26.18	25.48	2.216999
circSLC25A24(3)	chr1	108724563	108728576	SLC25A24	-	exon	5	8	26.58	25.41	1.856916
circFIRRE	chrX	130883334	130928494	FIRRE	-	exon	95	102	22.31	21.28	1.664765
circCDC25A(2)	chr3	48209336	48219478	CDC25A	-	exon	2	15	29.06	27.77	1.663955
circPLXNA4(3)	chr7	131908286	131913228	PLXNA4	-	exon	0	4	23.34	22.39	1.642703
circMYBL1	chr8	67513933	67514758	MYBL1	-	exon	4	14	18.96	17.91	1.520707
circSLC26A2	chr5	149357191	149361860	SLC26A2	+	exon	0	3	22.00	21.33	1.497724
circSGK223(2)	chr8	8233763	8239344	SGK223	-	exon	0	3	22.48	22.02	1.381539
circMAT1A	chr10	82039929	82043794	MAT1A	-	exon	0	2	19.35	18.84	1.368159
circKCNK5	chr6	39161945	39163763	KCNK5	-	exon	4	4	21.34	20.97	1.309321
circSLC25A19(1)	chr17	73279504	73282883	SLC25A19	-	exon	3	18	21.40	21.02	1.3029
circSLC25A24(4)	chr1	108697605	108728576	SLC25A24	-	exon	6	7	25.28	25.03	1.206263
circACOT2	chr14	74035982	74040294	ACOT2	+	exon	0	4	22.49	22.54	0.974017
circPLXNA4(2)	chr7	131844232	131853331	PLXNA4	-	exon	0	3	22.51	22.60	0.969025
circSGK223(1)	chr8	8196994	8215345	SGK223	-	intron	0	4	12.74	12.79	0.96619
circNR5A2	chr1	200080330	200090083	NR5A2	+	exon	0	4	23.42	23.55	0.934757
circZKSCAN1	chr7	99621042	99627998	ZKSCAN1	+	exon	15	23	23.20	22.99	0.862602

Figure S3

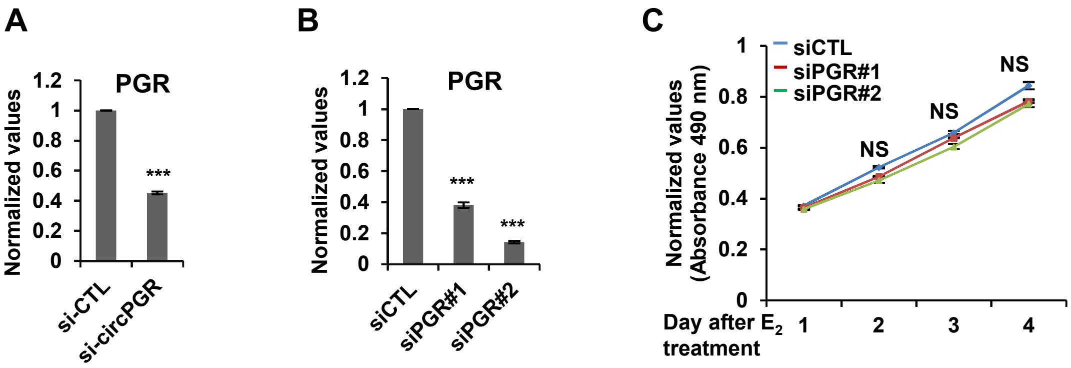
A
PGR
Normalized values
1.2
1
0.8
0.6
0.4
0.2
0
***
si-CTL
si-circPGR
B
PGR
Normalized values
***
***
siCTL
siPGR#1
siPGR#2
C
siCTL
siPGR#1
siPGR#2
Normalized values
(Absorbance 490 nm)
NS
NS
NS
Day after E2 treatment
1
2
3
4

Figure S4

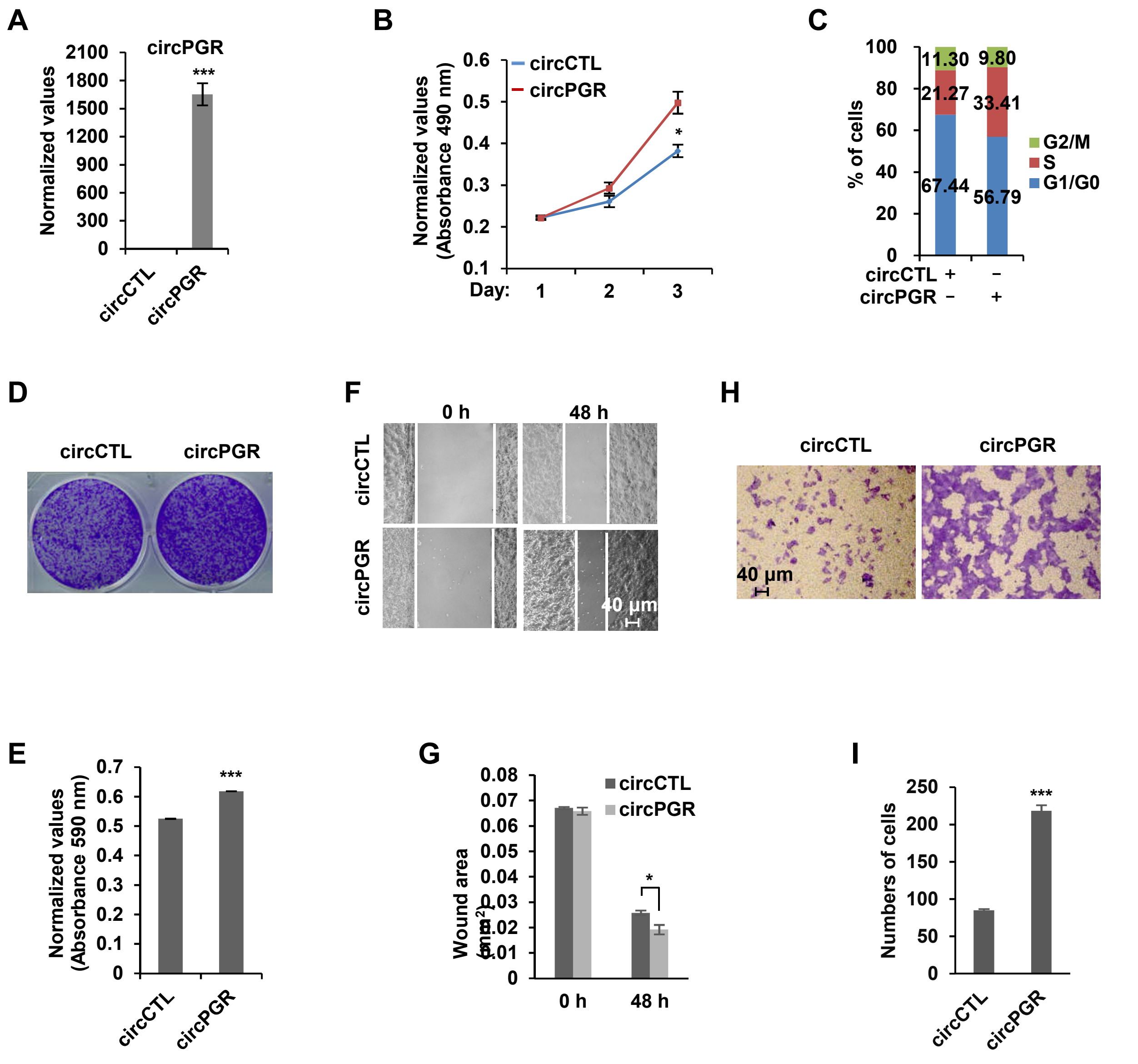
A
circPGR
Normalized values
2100
1800
1500
1200
900
600
300
0
***
circCTL
circPGR
B
Normalized values (Absorbance 490 nm)
circCTL
circPGR
0.6
0.5
0.4
0.3
0.2
0.1
*
Day: 1 2 3
C
% of cells
100
80
60
40
20
0
11.30
9.80
21.27
33.41
67.44
56.79
G2/M
S
G1/G0
circCTL + −
circPGR − +
D
circCTL
circPGR
F
0 h
48 h
circCTL
circPGR
40 μm
H
circCTL
circPGR
40 μm
E
Normalized values (Absorbance 590 nm)
0.7
0.6
0.5
0.4
0.3
0.2
0.1
0
***
circCTL
circPGR
G
Wound area (mm^2)
0.08
0.07
0.06
0.05
0.04
0.03
0.02
0.01
0
circCTL
circPGR
*
0 h
48 h
I
Numbers of cells
250
200
150
100
50
0
***
circCTL
circPGR

Figure S5

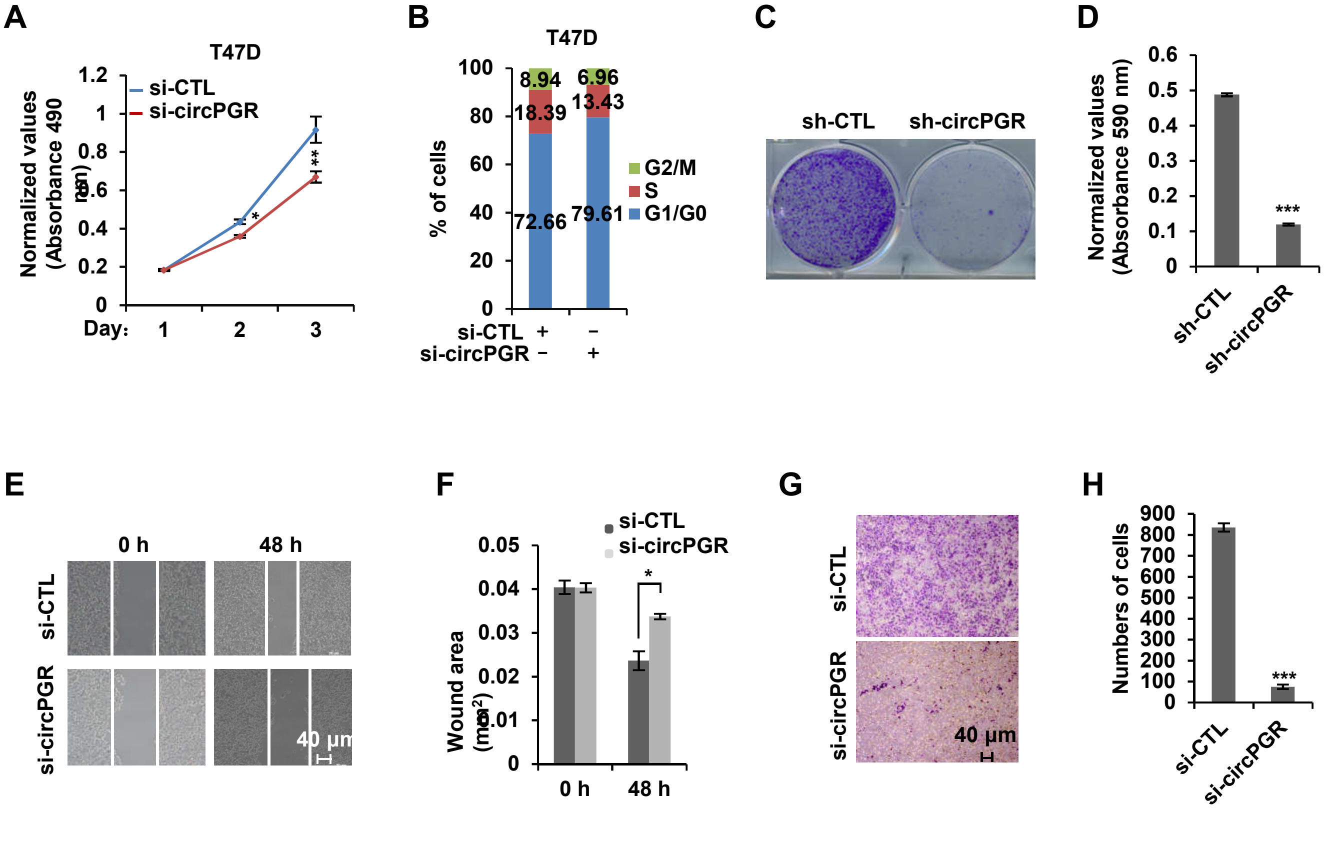
A
T47D
Normalized values (Absorbance 490 nm)
si-CTL
si-circPGR
Day:
B
T47D
% of cells
G2/M
S
G1/G0
8.94
18.39
72.66
6.96
13.43
79.61
si-CTL
si-circPGR
C
sh-CTL
sh-circPGR
D
Normalized values (Absorbance 590 nm)
sh-CTL
sh-circPGR
***
E
0 h
48 h
si-CTL
si-circPGR
40 μm
F
Wound area (mm²)
si-CTL
si-circPGR
0 h
48 h
G
si-CTL
si-circPGR
40 μm
H
Numbers of cells
si-CTL
si-circPGR
***

Figure S6

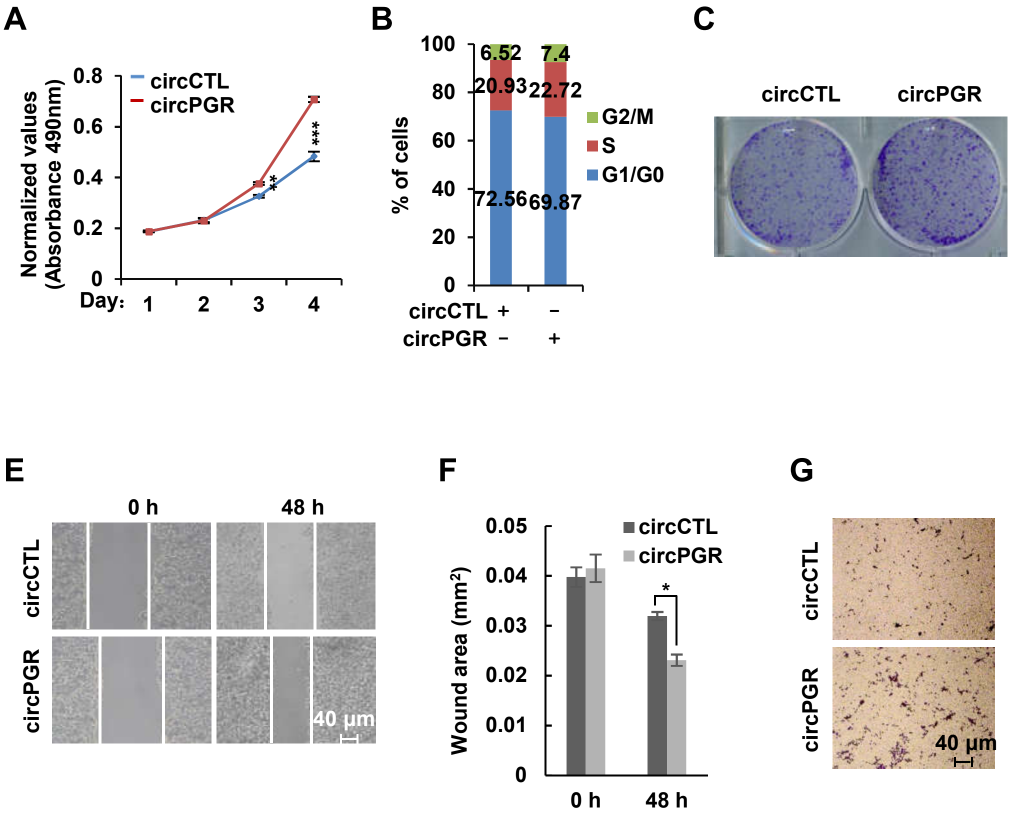
A
circCTL
circPGR
Normalized values (Absorbance 490nm)
Day: 1 2 3 4
B
% of cells
G2/M
S
G1/G0
6.52
7.4
20.93
22.72
72.56
69.87
circCTL + –
circPGR – +
C
circCTL
circPGR
D
Normalized values (Absorbance 590nm)
circCTL
circPGR
E
0 h
48 h
circCTL
circPGR
40 µm
F
Wound area (mm²)
circCTL
circPGR
0 h
48 h
G
circCTL
circPGR
40 µm
H
Numbers of cells
circCTL
circPGR

Figure S7

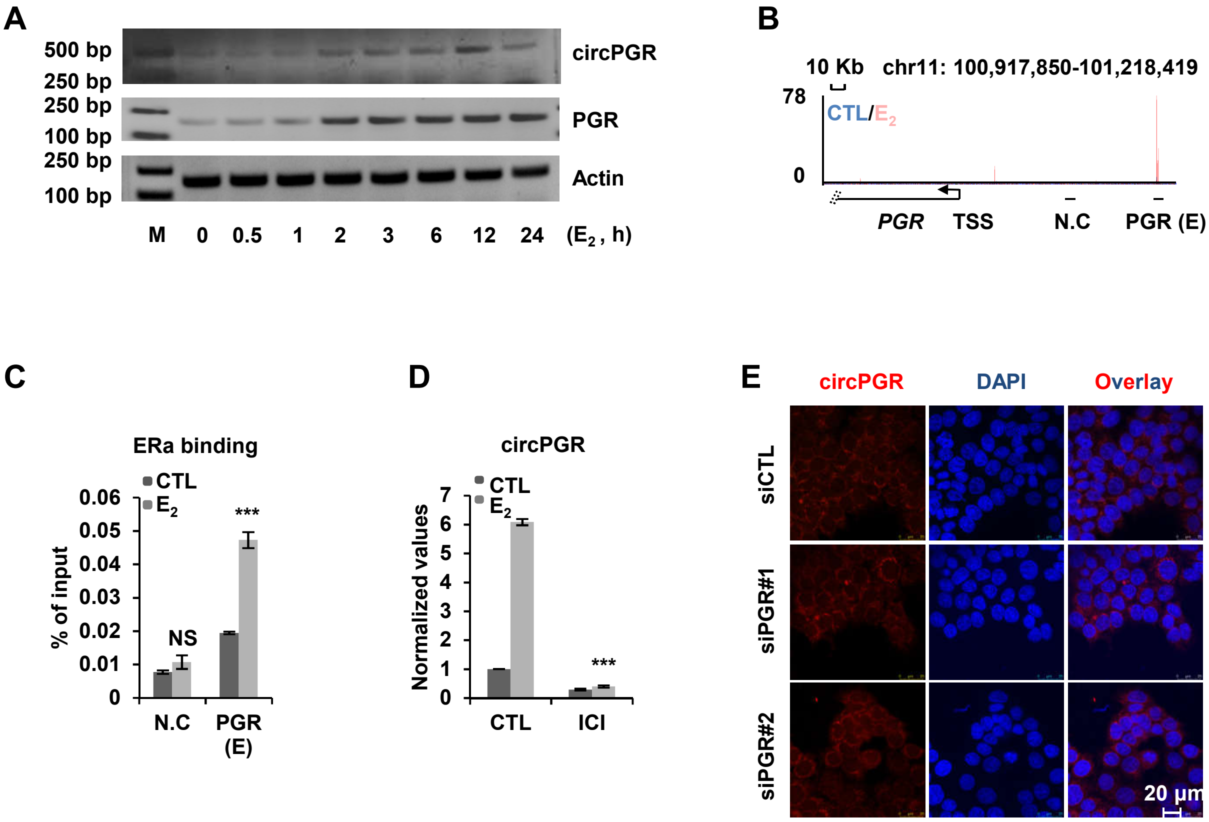
A
500 bp
250 bp
250 bp
100 bp
250 bp
100 bp
circPGR
PGR
Actin
M 0 0.5 1 2 3 6 12 24 (E2 , h)
B
10 Kb chr11: 100,917,850-101,218,419
78
0
CTL/E2
PGR TSS N.C PGR (E)
C
ERa binding
CTL
E2
% of input
0.06
0.05
0.04
0.03
0.02
0.01
0
NS
***
N.C
PGR (E)
D
circPGR
CTL
E2
Normalized values
7
6
5
4
3
2
1
0
***
CTL
ICI
E
circPGR
DAPI
Overlay
siCTL
siPGR#1
siPGR#2
20 µm

Figure S8

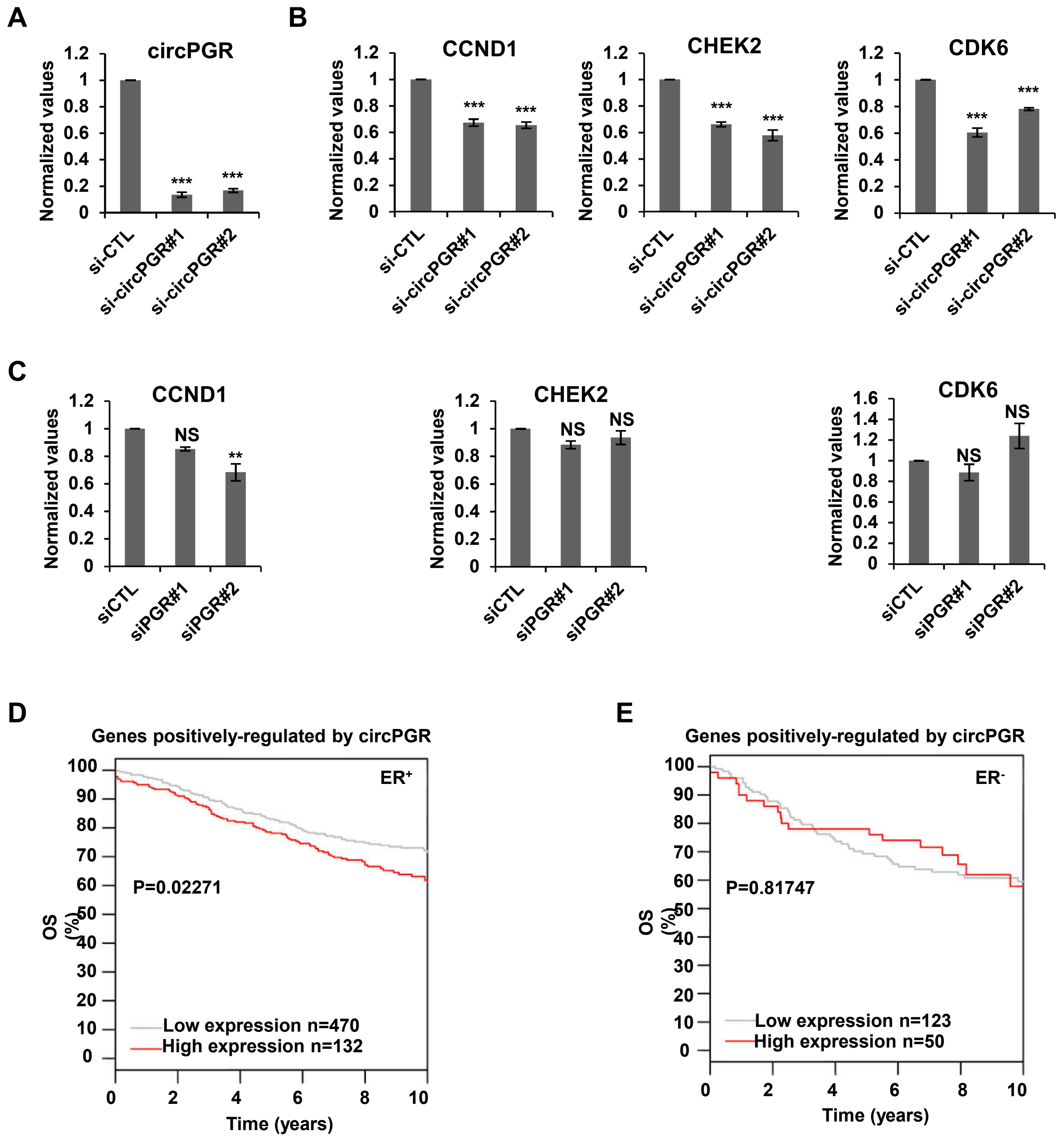

A
circPGR
Normalized values
si-CTL
si-circPGR#1
si-circPGR#2
***
B
CCND1
CHEK2
CDK6
C
CCND1
CHEK2
CDK6
siCTL
siPGR#1
siPGR#2
NS
**
D
Genes positively-regulated by circPGR
ER+
P=0.02271
OS (%)
Low expression n=470
High expression n=132
Time (years)
E
Genes positively-regulated by circPGR
ER-
P=0.81747
OS (%)
Low expression n=123
High expression n=50
Time (years)

Figure S9

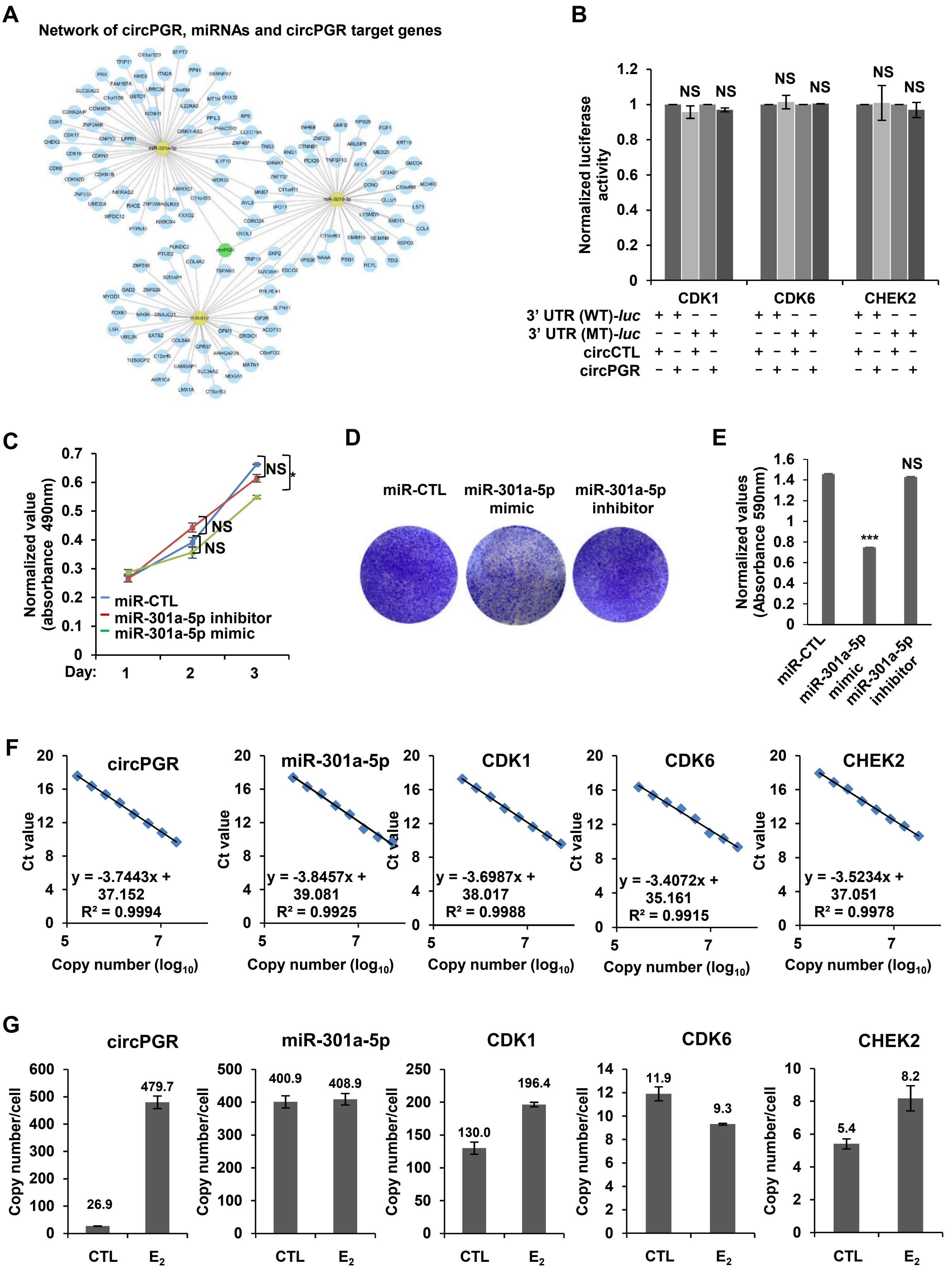
A
Network of circPGR, miRNAs and circPGR target genes
B
Normalized luciferase activity
NS
CDK1
CDK6
CHEK2
3' UTR (WT)-luc
3' UTR (MT)-luc
circCTL
circPGR
C
Normalized value (absorbance 490nm)
miR-CTL
miR-301a-5p inhibitor
miR-301a-5p mimic
Day:
D
miR-CTL
miR-301a-5p mimic
miR-301a-5p inhibitor
E
Normalized values (Absorbance 590nm)
***
F
circPGR
y = -3.7443x + 37.152
R² = 0.9994
miR-301a-5p
y = -3.8457x + 39.081
R² = 0.9925
CDK1
y = -3.6987x + 38.017
R² = 0.9988
CDK6
y = -3.4072x + 35.161
R² = 0.9915
CHEK2
y = -3.5234x + 37.051
R² = 0.9978
Ct value
Copy number (log10)
G
Copy number/cell
CTL
E2
26.9
479.7
400.9
408.9
130.0
196.4
11.9
9.3
5.4
8.2

Figure S10

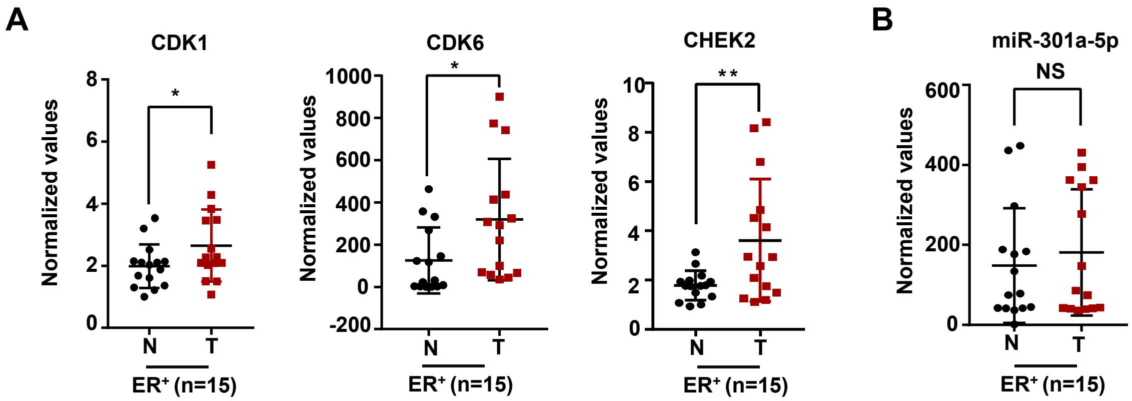

Figure S11
